# A Network of HMG-box Transcription Factors Regulates Sexual Cycle in the Fungus *Podospora anserina*

**DOI:** 10.1371/journal.pgen.1003642

**Published:** 2013-07-18

**Authors:** Jinane Ait Benkhali, Evelyne Coppin, Sylvain Brun, Leonardo Peraza-Reyes, Tom Martin, Christina Dixelius, Noureddine Lazar, Herman van Tilbeurgh, Robert Debuchy

**Affiliations:** 1 Université Paris-Sud, Institut de Génétique et Microbiologie UMR8621, Orsay, France; 2 CNRS, Institut de Génétique et Microbiologie UMR8621, Orsay, France; 3 Université Paris Diderot, Sorbonne Paris Cité, Institut des Energies de Demain (IED), Paris, France; 4 Department of Plant Biology and Forest Genetics, Uppsala BioCenter, Swedish University of Agricultural Sciences and Linnean Center for Plant Biology, Uppsala, Sweden; 5 Université Paris-Sud, Institut de Biochimie et de Biophysique Moléculaire et Cellulaire, UMR8619, Orsay, France; Duke University Medical Center, United States of America

## Abstract

High-mobility group (HMG) B proteins are eukaryotic DNA-binding proteins characterized by the HMG-box functional motif. These transcription factors play a pivotal role in global genomic functions and in the control of genes involved in specific developmental or metabolic pathways. The filamentous ascomycete *Podospora anserina* contains 12 HMG-box genes. Of these, four have been previously characterized; three are mating-type genes that control fertilization and development of the fruit-body, whereas the last one encodes a factor involved in mitochondrial DNA stability. Systematic deletion analysis of the eight remaining uncharacterized HMG-box genes indicated that none were essential for viability, but that seven were involved in the sexual cycle. Two HMG-box genes display striking features. *PaHMG5*, an ortholog of *SpSte11* from *Schizosaccharomyces pombe*, is a pivotal activator of mating-type genes in *P. anserina*, whereas *PaHMG9* is a repressor of several phenomena specific to the stationary phase, most notably hyphal anastomoses. Transcriptional analyses of HMG-box genes in HMG-box deletion strains indicated that *PaHMG5* is at the hub of a network of several HMG-box factors that regulate mating-type genes and mating-type target genes. Genetic analyses revealed that this network also controls fertility genes that are not regulated by mating-type transcription factors. This study points to the critical role of HMG-box members in sexual reproduction in fungi, as 11 out of 12 members were involved in the sexual cycle in *P*. *anserina*. PaHMG5 and SpSte11 are conserved transcriptional regulators of mating-type genes, although *P. anserina* and *S. pombe* diverged 550 million years ago. Two HMG-box genes, *SOX9* and its upstream regulator *SRY*, also play an important role in sex determination in mammals. The *P. anserina* and *S. pombe* mating-type genes and their upstream regulatory factor form a module of HMG-box genes analogous to the SRY/SOX9 module, revealing a commonality of sex regulation in animals and fungi.

## Author Summary

*Podospora anserina*, a coprophilous fungus, is used extensively as a model organism to address questions of sexual development and mating-type functions. Its mating-type locus contains three HMGB genes that encode transcription factors involved in fertilization and fruit-body development. We present the functional characterization of the remaining HMGB genes, which revealed that 11 of 12 HMGB genes were involved in sexual development. An analysis of the relationships between these genes uncovered a regulatory network governing the expression of mating-type genes. PaHMG5 is a key transcription factor that operates upstream of mating-type genes in this network. A homolog of PaHMG5 performs a similar function in the fission yeast *Schizosaccharomyces pombe*, which diverged from *P*. *anserina* 550 million years ago. The conservation of a regulatory circuit over such a prolonged timeframe is a striking exception to the general observation that sex developmental pathways are highly variable, even across closely related lineages. A module consisting of two HMGB transcription factors (Sry and Sox9) is a key regulator of sex determination in mammals. We propose that the module containing *PaHMG5* and mating-type HMGB genes is the fungal counterpart of the mammalian module, revealing a commonality of sex regulation in animals and fungi.

## Introduction

High-mobility-group box (HMGB) proteins [1] include chromatin architectural proteins as well as specific transcription factors that are involved in highly diverse functions ranging from sex determination [2] to extracellular immune signaling [3], [4]. All of these functions rely on the HMG-box, an HMGB conserved motif containing approximately 80 amino acids arranged in a distinctive L-shaped three-α-helical fold [5], [6]. This HMG-box motif sharply bends DNA [7] and facilitates the assembly of transcriptional complexes that involve other proteins by distorting chromatin [8]. Based on phylogenetic analyses of the HMG-box, the HMGB superfamily can be divided into two families, HMGB-UBF_HMG and SOX/TCF/MATA_HMG [9]. These families were named after the best known representative in each group, namely hUBF [10], the SOX (Sry-type HMG-box) genes [11], TCF-1 [12] and MATa-1 [13]. The HMGB-UBF_HMG family is considered to be sequence non-specific, as generic UBF proteins can bind both ribosomal DNA regulatory sequences and sequences across the entire ribosomal DNA repeat [14]. Members of this family are present in plants, fungi and animals. On the other hand, the SOX/TCF/MATA_HMG family contains proteins that bind specific DNA sequences, with a common T/A rich core (reviewed in [2]). The SOX/TCF/MATA_HMG family is subdivided into the SOX-TCF_HMG and the MATA_HMG subfamilies. The MATA_HMG subfamily includes exclusively fungal proteins, most of which are involved in sexual processes, while SOX-TCF_HMG genes appear to be restricted to animals [9].

Members of the HMGB-UBF_HMG family function in many processes such as transcription, genomic stability and the three R's (replication, recombination and DNA repair) (reviewed in [8]). An exhaustive analysis of HMGB-UBF_HMG_box encoding genes was performed in *Saccharomyces cerevisiae* in a previous study, and it showed HMGB-UBF_HMG-box genes to have highly diversified functions. In brief, the six HMGB-UBF_HMG_box genes from *S. cerevisiae* are involved in ribosomal DNA transcription [15], chromatin remodeling complexes [16], [17], and mitochondrial metabolism and energy [18], [19], [20]. Analysis of HMGB-UBF_HMG-box genes in *S. pombe* mainly focused on the *splsd1* and *splsd2* genes. The SpLsd1 protein functions as a histone demethylase, and both SpLsd1/2 are believed to affect the epigenetic state of the cell [21].

Members of the SOX/TCF/MATA_HMG family are important regulators of differentiation and the sexual process. In mammals, the Y-linked testis-determining factor, SRY, is the founding member of the SOX subfamily [11]. SRY induces male sex determination by regulating *Sox9* [22], [23], [24]. Unlike *Sry*, *Sox9* is conserved among non-mammalian vertebrates and induces male-to-female sex-reversal when mutated [25], illustrating that it is ancestral and critical for vertebrate function. HMG-box genes also play a central role in the fungal mating process. Almost all mating-type loci from Ascomycota contain at least one MATA_HMG gene [26], [27]. The most notable exception to this is *S*. *cerevisiae* (Saccharomycotina), which lost one MATA_HMG mating-type gene during evolution [28] but retained the prototypical α1 protein MATα1p. MATα1p was identified as a member of the MATA_HMG subfamily [27]. *ROX1* is the other gene of this subfamily in *S. cerevisiae* and one of the few fungal MATA_HMG-box genes that are not involved in sexual reproduction. Instead, it represses the expression of hypoxic genes [29]. In *S. pombe*, mating-type gene transcription is regulated by the MATA_HMG-box gene, *SpSte11* [30]. The activation of the sexual process in *S*. *pombe* relies on the nuclear accumulation of SpSte11, which is triggered by starvation and pheromone signaling [31]. An allelic system of HMG-box genes that putatively determine mating types was also identified in Zygomycota [32], [33], [34], [35] and Microsporidia [34], [36], but they were part of the SOX-TCF_HMG and HMGB-UBF_HMG groups [27]. In Basidiomycota, mating-type loci do not contain HMG-box genes, but MATA_HMG-box genes play an essential role in sexual development, as shown in *Ustilago maydis* [37], [38], [39], *Coprinopsis cinerea* [40] and *Cryptococcus neoformans* [41], [42].

A genome-wide systematic deletion analysis of transcription factors performed in the homothallic fungus *Fusarium graminearum* indicated that several HMG-box genes are involved in sexual development [43]. However, not all HMG-box genes from *F*. *graminearum* were deleted in that study and their genetic relationships were not investigated. Here, we report the systematic deletion analysis of HMG-box genes in the heterothallic fungus *Podospora anserina* as well as an in-depth analysis of their genetic interactions. The *P. anserina* genome encodes a total of 12 HMG-box genes. Of these, four have been previously characterized, including three mating-type genes (*FPR1*, *FMR1* and *SMR2*) belonging to the MATA_HMG subfamily [27], and the nuclear gene encoding a mitochondrion-targeted HMGB-UBF-box protein (*mtHMG1, Pa_1_13340*) [44] (Table 1). *FPR1* is encoded by the *mat+* idiomorph [45], while *FMR1* and *SMR2* are both encoded by the *mat-* idiomorph [45], [46]. These mating-type genes control fertilization and the development of the fruit-body (reviewed in [47]). The remaining eight uncharacterized HMG-box genes encode two MATA_HMG-box proteins, four HMGB-UBF_HMG-box proteins, and two proteins that contain an HMG-box with an atypical residue. In the present study, these genes were individually deleted and the phenotypes of mutant strains were carefully assessed. The results revealed that all genes were dispensable for viability. Moreover, six mutant strains displayed sexual reproduction defects, and one of the two remaining mutants displayed vegetative defects. Transcriptional analyses indicated that an ortholog of *SpSte11* is at the hub of the HMG-box gene network and that it regulates the expression of *FMR1* and *FPR1* and fertility.

**Table 1:** HMG-box genes in *P. anserina*.

Gene number (gene name)	Gene function	HMGB family	Additional domain		Orthologs	
				*S. cerevisiae*	*S. pombe*	*N. crassa*
*(FMR1)*	Mating-type regulation	MATA_HMG	-	*MATα1*	-	*NCU01958*
*(SMR2)*	Mating-type regulation	MATA_HMG	-	-	-	*NCU01960*
*Pa_1_20590 (FPR1)*	Mating-type regulation	MATA_HMG	-	-	*Mc*	*MAT a-1*
*Pa_1_13340 (mtHMG1)*	Mitochondrial DNA stability	HMGB_UBF	DUF1898	-	-	*NCU02695*
*Pa_1_7390 (PaHMG2)*	Unknown function	HMGB_UBF	SAM	-	-	*NCU02819*
*Pa_1_9380 (PaHMG3)*	Unknown function	HMGB_UBF	SWIRM, amine-oxidase	-	*lsd1*	*NCU09120*
*Pa_1_11050 (PaHMG4)*	Unknown function	HMGB_UBF	-	-	-	*NCU03126*
*Pa_1_13940 (PaHMG5)*	Unknown function	MATA_HMG	-	-	*ste11*	*NCU02326 NCU09387*
*Pa_1_14230 (PaHMG6)*	Unknown function	HMGB_UBF	-	*NHP6B*	*NHP6*	*NCU09995*
*Pa_5_8400 (PaHMG7)*	Unknown function	HMGB	SprT-like	-	*NP_595970.1*	*NCU06874*
*Pa_6_4110 (PaHMG8)*	Unknown function	MATA_HMG	-	*ROX1*	-	*NCU03481*
*Pa_7_7190 (PaHMG9)*	Unknown function	HMGB	-	-	-	*NCU07568*

## Results

### Identification of HMG-box genes in *P*. *anserina*

**Figure 1:**
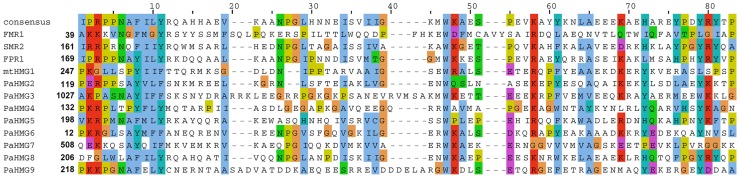
Alignment of HMG domains from the 12 HMG-box proteins of *P. anserina*. The alignment was performed using ClustalW2 [105] and colored according to the Clustal X color scheme provided by Jalview [106]. This color scheme is displayed in Table S1.

The Podospora protein database (Materials and Methods) was searched for HMGB proteins using the previously defined HMG-box consensus sequence [27] as query in a Blastp search [48]. *P. anserina* genome annotation in Fungal Transcription Factor [49] and Superfamily databases [50] was examined to identify possible missing HMGB genes in our analyses. A total of 12 HMG-box genes were identified. Of these, eight had not been previously characterized and were here named *PaHMG2* to *PaHMG9* (Table 1). CD-Search [51] identified five MATA_HMG-box proteins and five HMGB-UBF_HMG-box proteins (Table 1), but failed to categorize two HMG-box proteins. These two proteins, PaHMG7 and PaHMG9, contained an atypical charged residue instead of a conserved aliphatic or aromatic amino acid at position 9 (Figure 1). However, they contained the specific aromatic amino acids that anchor the recognition helix of the HMG-box domain to the hydrophobic core at position 8 and 11, confirming that they belong to the HMGB superfamily. Additional domains are shown in Table 1. Except for mtHMG1, which contained a mitochondrial targeting signal, the remaining 11 HMG-box proteins from *P*. *anserina* were predicted to localize to the nucleus.

A search for orthologs of *P*. *anserina* HMG-box genes with FUNGIpath [52] in selected fungal species indicated that three had an ortholog in *S*. *cerevisiae*, six had an ortholog in *S*. *pombe*, and all had an ortholog in *Neurospora crassa* (Table 1). PaHMG6, PaHMG7 and PaHMG8 had orthologs in Basidiomycota but the functions of these orthologs have not been investigated yet. *Prf1*, *Rop1* and *HMG3* from *U*. *maydis* [37], [38], *Mat2* from *C*. *neoformans* [42] and *Pcc1* from *C*. *cinerea* [40] encoded HMG-box regulators that are related to the sexual cycle in Basidiomycota. FUNGIpath did not detect any *P*. *anserina* orthologs for these HMG-box genes.

Most HMG-box genes localized to chromosome 1, which contains the mating-type locus [53]. Only three out of 12 HMGB genes mapped outside of chromosome 1, suggesting that the distribution of HMG-box genes may be biased to chromosome 1. However, statistical tests indicated this bias to be inconclusive (Material and Methods).

### Evolutionary analysis of fungal HMG-box proteins

**Figure 2:**
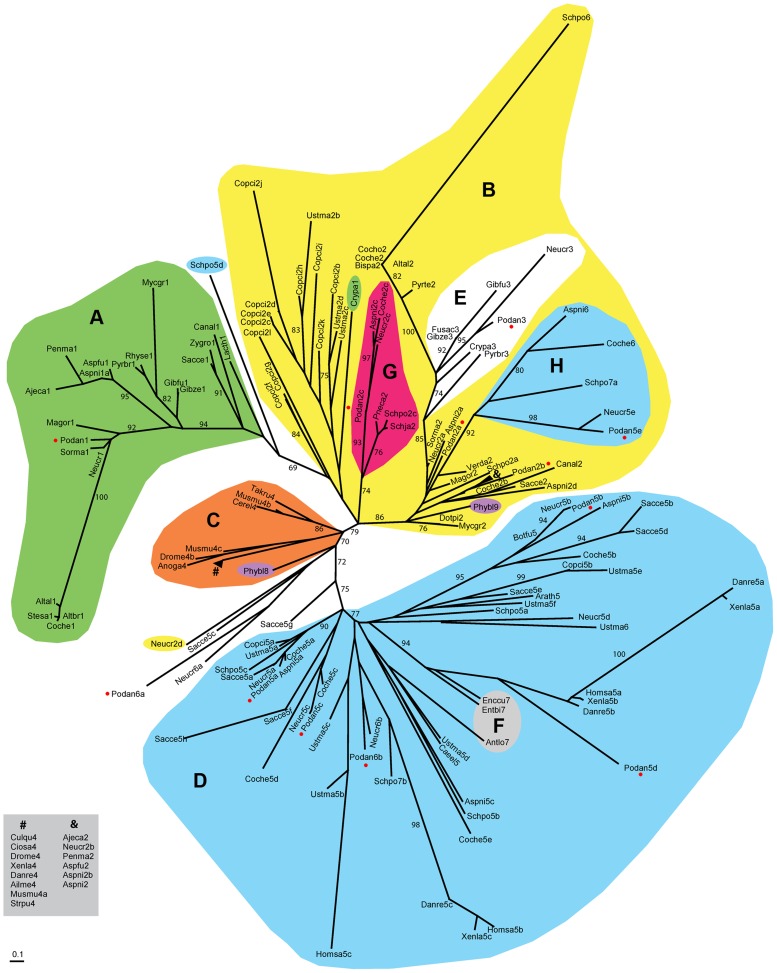
Unrooted phylogram for the HMG-box superfamily. Clustering of core amino acid sequences using maximum-likelihood and model LG+G [107]. Color labeling: MATα_HMG (A, green), MATA_HMG (B, yellow), SOX-TCF_HMG (C, orange), HMGB-UBF_HMG (D, H, blue), MAT1-1-3 in MATA_HMG (E, white) and STE11 in MATA_HMG (G, red). Other labels: Microsporidia MAT sex locus in HMGB-UBF_HMG (F, grey), *Phycomyces blakesleeanus* (Zygomycota) sexM (Phybl8) and sexP (Phybl9) (purple) and *P. anserina* proteins (red dots). LR-ELW values greater than 70% are shown. Abbreviations: *Ailuropoda melanoleuca* (Ailme); *Ajellomyces capsulatus* (Ajeca); *Alternaria alternata* (Altal); *Alternaria brassicicola* (Altbr); *Anopheles gambiae* (Anoga); *Antonospora locustae* (Antlo); *Arabidopsis thaliana* (Arath); *Aspergillus fumigatus* (Aspfu); *Aspergillus nidulans* (Aspni); *Botryotinia fuckeliana* (Botfu); *Bipolaris sacchari* (Bipsa); *Caenorhabditis elegans* (Caeel); *Candida albicans* (Canal); *Cervus elaphus yarkandensis* (Cerel); *Ciona savignyi* (Ciosa); *Cochliobolus heterostrophus* (Coche); *Cochliobolus homomorphus* (Cocho); *Coprinopsis cinerea* (Copci); *Cryphonectria parasitica* (Crypa); *Culex quinquefasciatus* (Culqu); *Danio rerio* (Danre); *Dothistroma pini* (Dotpi); *Drosophila melanogaster* (Drome); *Enterocytozoon bieneusi* (Entbi); *Encephalitozoon cuniculi* (Enccu); *Fusarium acaciae-mearnsii* (Fusac); *Gibberella fujikuroi* (Gibfu); *Gibberella zeae* (Gibze); *Homo sapiens* (Homsa); *Lachancea thermotolerans* (Lacth); *Magnaporthe oryzae* (Magor); *Mycosphaerella graminicola* (Mycgr); *Podospora anserina* (Podan); *Neurospora crassa* (Neucr); *Penicillium marneffei* (Penma); *Phycomyces blakesleeanus* (Phybl); *Pneumocystis carinii* (Pneca); *Pyrenopeziza brassicae* (Pyrbr); *Pyrenophora teres* (Pyrte); *Rhynchosporium secalis* (Rhyse); *Saccharomyces cerevisiae* (Sacce); *Schizosaccharomyces japonicus* (Schja); *Schizosaccharomyces pombe* (Schpo); *Sordaria macrospora* (Sorma); *Stemphylium sarciniforme* (Stesa); *Strongylocentrotus purpuratus* (Strpu); *Takifugu rubripes* (Takru); *Ustilago maydis* (Ustma); *Verticillium dahliae* (Verda); *Xenopus laevis* (Xenla); and *Zygosaccharomyces rouxii* (Zygro). Numbers after species names indicate α1 proteins (1), MATA_HMG (2), MAT1-1-3 (3), SOX (4), HMGB-UBF_HMG (5) and other HMG domains (6–9). When more than one domain was present for the same species, the suffix a, b or c was used. Units indicate the number of amino acid changes per position. Species codes and accession numbers grouped by evolutionary affinity are listed in Table S2.

The HMG-box of the 12 *P*. *anserina* HMGB proteins was extracted and grouped in a phylogram with the HMG-box motif of selected plant, animal and fungal species, including *S. cerevisiae*, *S. pombe*, *N. crassa*, *Aspergillus nidulans*, *Cochliobolus heterostrophus* and *U. maydis* (Figure 2). A total of 154 HMG-box domains were clustered in four groups, which overall corresponded to the previously defined HMGB groups [9]: MATα_HMG (groupe A, Figure 2) [27], MATA_HMG (group B, Figure 2), SOX-TCF_HMG (group C, Figure 2) and HMGB-UBF_HMG (group D, Figure 2).

Group A (Figure 2) included exclusively the mating-type transcription factors with an α1 domain, which was proposed to correspond to a new class of HMG-box [27], [54]. Group A formed a clade related to the MATA_HMG subfamily and contained FMR1 (Podan1). The other HMG-box mating-type transcription factors FPR1 (Podan2a) and SMR2 (Podan3) were placed in group B (Figure 2), which contained mostly MATA_HMG-box proteins. PaHMG5 (Podan2c) clustered within clade G, with SpSte11 (Schpo2c) of *S*. *pombe* [30] and NCU09387/FMF-1 (Neucr2c) of *N*. *crassa* [55], [56]. The orthology of these three proteins, suggested by their phylogenic position, was independently confirmed with FUNGIpath. Moreover, the relationship of NCU09387 (Neucr2c) with SpSte11 (Schpo2c) was previously reported [57]. PaHMG8 (Podan2b) was the only MATA_HMG-box protein encoded by a gene outside chromosome 1. Its ortholog in *S*. *cerevisiae* is ROX1p (Sacce2), a repressor of hypoxic genes, and one of the few MATA_HMG-box proteins not related to mating processes [29]. Earlier evolutionary analysis of the oxygen-responding system in *Kluyveromyces lactis* and *S. cerevisiae* suggested that *ROX1* was recruited specifically to control this system in *S. cerevisiae* [58]. Rfg1p (Canal2), the ortholog of ROX1p (Sacce2) in *Candida albicans*, controls filamentous growth and virulence [59]. Interestingly, the clade defined by PaHMG8 (Podan2b), ROX1p (Sacce2) and Rfg1p (Canal2) included a MAT1-2-1 mating-type protein (Ajeca2, see ‘&’ in clade B, Figure 2) and was placed close to the MAT1-2-1 (Podan2a, Aspni2a, Neucr2a and Sorma2) and MAT1-1-3 mating-type proteins (clade E, Figure 2). The placement of the PaHMG8 (Podan2b)/ROX1p (Sacce2) clade in the MATA_HMG subfamily and our functional analyses of PaHMG8 (Podan2b) (see below) supports the idea that the ancestral *ROX1* gene was primarily involved in sexual development before being recruited for various other functions in *S*. *cerevisiae* and *C*. *albicans*. Surprisingly, group B contained an HMGB-UBF_HMG clade (clade H, Figure 2), which included PaHMG3 (Podan5e) and several fungal orthologs, notably the histone demethylase SpLsd1 (Schpo7a) of *S*. *pombe* [21]. All proteins from clade H have SWIRM and amino oxydase domains, which are characteristic of histone demethylases, thus supporting their orthologous relationship.

Five *P*. *anserina* HMG-box proteins were classified into group D (Figure 2), which comprised members of the HMGB-UBF_HMG family, except the clade grouping PaHMG7 (Podan6b), NCU06874 (Neucr6b) from *N*. *crassa* and NP_595970 (Schpo7b) from *S*. *pombe*. These three proteins are characterized by a conserved atypical residue in the HMG-box domain [see the above section for its description in PaHMG7 (Podan6b)] and a SprT-like domain (Interpro accession number IPR006640). The four HMGB-UBF_HMG-box proteins, PaHMG2 (Podan5c), PaHMG4 (Podan5b), mtHMG1 (Podan5d), and PaHMG6 (Podan5a), branched into different clades of group D. PaHMG2 (Podan5c) belonged to a branch that included proteins with a Sterile Alpha Domain (SAM), which is involved in protein-protein and protein-RNA interactions [60]. In some cases, the orthologous relationships, as defined by FUNGIpath, were not in agreement with the HMG-box based phylogeny. The first instance was the mitochondrial protein mtHMG1 (Podan5d) and its ortholog NCU02695 (Neucr5d) in *N*. *crassa*, which failed to group into the same clade within the HMGB-UBF_HMG family although FUNGIpath provided a high confidence score for the orthology of mtHMG1 (Podan5d) and NCU02695 (Neucr5d) HMG-box domain. Moreover, mtHMG1 (Podan5d) and NCU02695 (Neucr5d) have a mitochondrial targeting signal and are characterized by a DUF1898 domain, supporting an orthologous relationship. In another instance, Nhp6Ap (Sacce5a) and Nhp6Bp (Sacce5b), two functionally redundant putative inparalogs from *S*. *cerevisiae* collectively referred to as Nhp6p [61], [62], were placed in distant clades with PaHMG6 (Podan5a) and PaHMG4 (Podan5b), respectively, and may be outparalogs instead. In contrast, FUNGIpath identified Nhp6p (Sacce5a, b) as co-orthologs of PaHMG6 (Podan5a). Similarly, Hmo1p (Sacce5d) from *S*. *cerevisiae* belonged to the same clade as PaHMG4 (Podan5b), while FUNGIpath identified Hmo1p (Sacce5d) as an ortholog of PaHMG6 (Podan5a). However, the FUNGIpath confidence score for orthology of PaHMG6 (Podan5a), Nhp6p (Sacce5a, b), and Hmo1p (Sacce5d) was low. Further analyses will be necessary to resolve these phylogenetic ambiguities.

PaHMG9 (Podan6a), along with its *N. crassa* ortholog NCU07568 (Neucr6a), were placed between group C and D (Figure 2). Accordingly, CD-Search failed to place PaHMG9 (Podan6a) and NCU07568 (Neucr6a) into either the MATA_HMG or HMGB-UBF groups (Table 1). Interestingly, the PaHMG9 HMG-box (Podan6a) is characterized by an atypical residue (see above section) that is conserved in NCU07568 (Neucr6a). Our phylogenic analysis also placed a MATA_HMG-box protein from *N. crassa* (NCU02326, Neucr2d) between group C and D (Figure 2). Previous work reported that NCU02326 (Neucr2d) is related to SpeSte11 (Schpo2c) and is the closest homolog to NCU09387 (Neucr2c) [57]. In agreement with this report, FUNGIpath identified NCU02326 (Neucr2d) as an ortholog of SpSte11 (Schpo2c, clade G in Figure 2) and an inparalog of NCU09387 (Neucr2c). However, unlike NCU09387 (Neucr2c), NCU02326 (Neucr2d) was not placed within clade G with all other SpSte11 orthologs. Such inconsistency is not unprecedented, as there are several evidences in *N*. *crassa* that effective defense against duplicated sequences prevents the maintenance of closely related paralogs in this species [63]. To independently assess which of the two *N. crassa* inparalogs is conserved in *P. anserina*, we compared the environment of these genes in the two species. The shared synteny observed upstream and downstream of *PaHMG5* (Podan2c) and *NCU09387* (Neucr2c) (Figure S1A) confirms that these two genes are orthologs. The absence of conserved organization between the *NCU02326* (Neucr2d) locus and its putative counterpart in *P. anserina* indicates that this gene is absent in *P. anserina* (Figure S1B).

### Phenotypic analysis of HMG-box deletion strains during vegetative growth

The eight additional HMG-box genes identified in this study were inactivated by targeted gene deletion to assess their role in the life cycle of *P. anserina*. Deletions were verified by Southern blot analysis (Materials and Methods). The eight mutants were examined for growth and for macroscopic mycelium alterations on minimal medium in *mat +* and *mat−* context. The *ΔPahmg2*, *ΔPahmg6* and *ΔPahmg9* strains displayed reduced growth (Table 2). The eight mutants were also tested for cold- and thermo-sensitive growth at 18°C and 36°C and for sensitivity to caffeine (phosphodiesterase inhibitor), fludioxonil, which acts on osmoregulation, and sodium dodecylsulfate, which acts on the cell membrane, without revealing any additive phenotype.

**Table 2:** Phenotypes of the *P. anserina* mutants with deleted HMG-box genes.

	Vegetative phenotype		Sexual phenotype		
Strain	Growth	Mycelium aspect	Fertility^a^	Number of spermatia	Distribution of perithecia^b^
*ΔPahmg2*	90% of wild-type	pigmented	male and female fertile	wild-type	ring as wild type
*ΔPahmg3*	wild-type	wild-type	male and female fertile	wild-type	ring ø = 53.7 mm±0,9
*ΔPahmg4*	wild-type	wild-type	male and female fertile	×4 to 7	no ring
*ΔPahmg5*	wild-type	wild-type	male and female sterile	wild-type	no perithecium
*ΔPahmg6*	90% of wild type	wavy	low female fertile, male fertile	wild-type	100 perithecia
*ΔPahmg7*	wild-type	wild-type	male and female fertile	wild-type	ring ø = 50.1 mm±1
*ΔPahmg8*	wild-type	wild-type	female sterile, male fertile	wild-type	no perithecium
*ΔPahmg9*	50% of wild type	flat and wavy	female sterile, male fertile	×10 to 20	no perithecium
*ΔPahmg3 ΔPahmg7*	wild-type	wild-type	male and female fertile	wild-type	ring ø = 54.9 mm±2.1
*wild type*	wild-type	wild-type	male and female fertile	wild-type	ring ø = 49.1 mm±1.8

a see Figure 4 A.

b see Figure 4 B. ø: outside diameter of the ring in mm (mean of measurements made in eight independent cultures).

**Figure 3:**
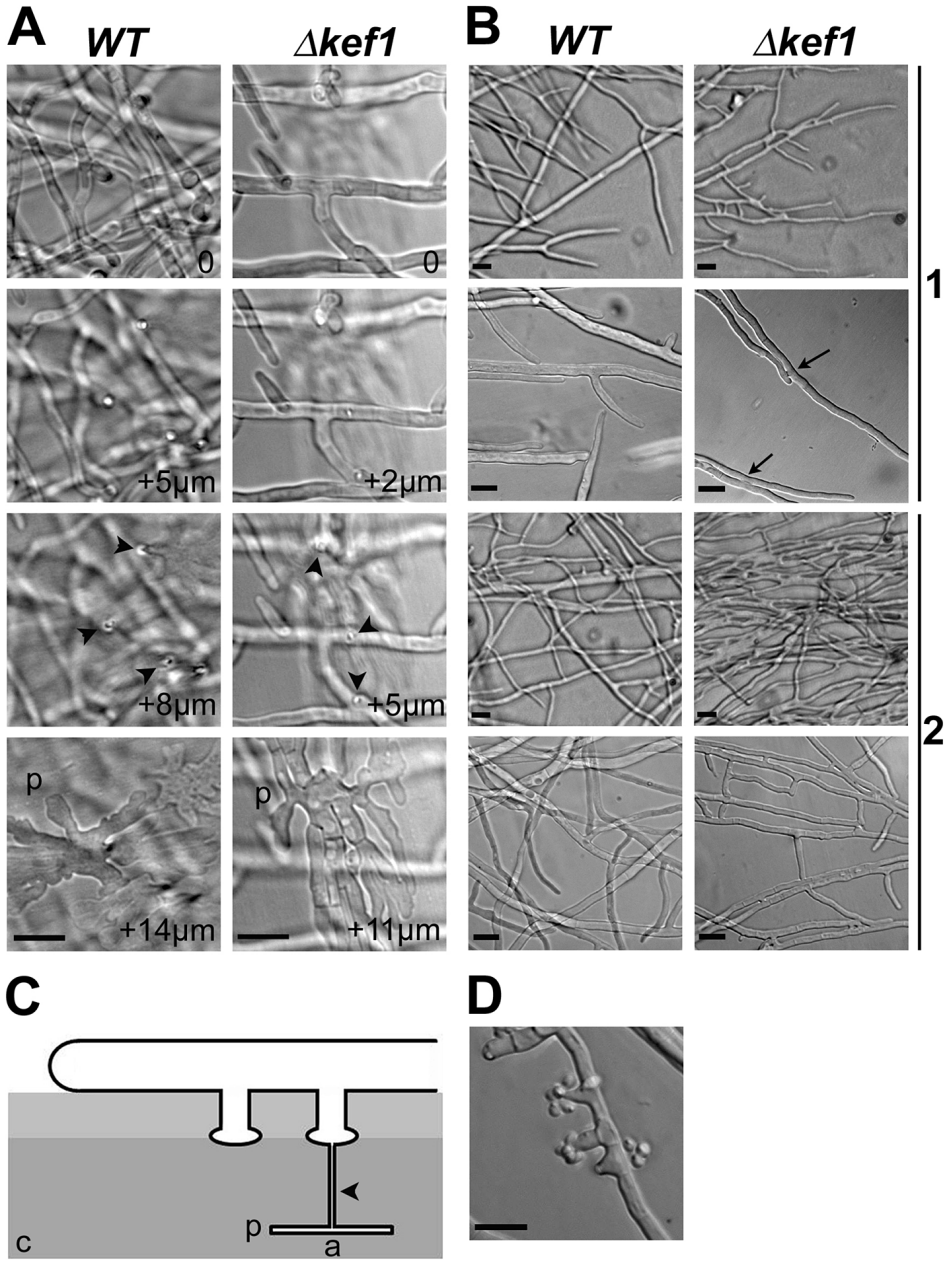
Microscopic phenotypes of *Δkef1* (*ΔPahmg9*) strain. (A) Appressorium-like structures in the *Δkef1* strain and in the wild type (*WT*). Development of appressorium-like structures was not affected in the *Δkef1* strain. The pictures were taken every 1 µm as Z stacks, with “0” corresponding to hyphae growing onto the cellophane layer (see panel C). Arrowheads indicate needle-like hyphae that penetrated the cellophane layer; p: palm-like structures within the cellophane layer. (B) Comparison of hyphal fusion (anastomosis) in wild-type (*WT*) and *Δkef1* strains. In the wild type, anastomoses were never observed in apical hyphae (1), while they were occasionally observed in the subapical area (2). In *Δkef1* strain, anastomoses were profuse in apical (1) and in subapical (2) areas. Most notably, anastomosis occurred between the apex of hyphae at the leading edge and neighbouring apical hyphae (see arrows in *Δkef1* in 1). (C) Schematic of appressorium-like development in *P. anserina*. a: appressorium-like structure including the palm-like structure and the needle-like hyphae; c: cellophane layer on which mycelium is growing. (D) Spermatia and their spermatogonia were present in the subapical area of the *Δkef1* strain but were absent from the same area in the wild-type strain. Scale bar = 10 µm in all panels.

Microscopic examination of a 4-day-old *ΔPahmg9* thallus revealed that the leading edge (apical and sub-apical hyphae) displayed several developmental processes that typically occur in old hyphae in the wild-type strain. For instance, male gametes (spermatia) were observed in the sub-apical portion of a *ΔPahmg9* thallus (Figure 3). *P. anserina* differentiates special cell structures dedicated to breach and to exploit solid cellulosic substrates like cellophane [64]. These structures resemble appressoria, the specialized infective structures of fungal plant pathogens. The *P. anserina* appressorium-like structures were identical in wild-type and *ΔPahmg9* strains (Figure 3A). However, they emerged sooner at the leading edge in the mutant strain than in the wild-type strain. The first appressorium-like structures were found at 1.58±0.31 mm and 4.08±0.20 mm from the hyphal tip in the *ΔPahmg9* and wild-type strains, respectively. Finally, the most striking phenotype of the *ΔPahmg9* strain was the dramatic increase in hyphal anastomoses. While few anastomoses are usually observed in the sub-apical portion of a wild-type thallus (Figure 3B), sub-apical hyphae from the *ΔPahmg9* strain fused with each other, leading to bundles of tightly attached hyphae (Figure 3B). Moreover, apical hyphae often fused with each other in the *ΔPahmg9* strain (Figure 3B). We never observed wild-type apical hypha anastomoses, suggesting that the *ΔPahmg9* mutation deregulates cell fusion. We therefore named this gene *KEF1* (KEep-on-Fusing 1). To our knowledge, *KEF1* is the first identified gene to have a repressor function during cell fusion, whereas many genes activate this process in *N*. *crassa* [65], [66] and *P. anserina* [67], [68] (reviewed in [69]).

### Phenotypic analysis of HMG-box deletion strains during sexual development

*P. anserina* is a heterothallic (self-sterile) fungus that has two mating types, *mat+* and *mat−*. Haploid strains of each mating type initiate the sexual cycle by differentiating male gametes (spermatia) and female organs (protoperithecia). A phenotypic analysis of sexual development separately evaluates the male and female fertility of each mutant. The female organ is a multicellular structure comprising protective maternal hyphae and the ascogonium, which contains the female gametic nuclei. The spermatia are independent cells that can be collected and used to fertilize any strain of opposite mating type. A pheromone/receptor signaling system allows the ascogonium to recognize and fuse with sexually compatible spermatia. Therefore, fertilization can be controlled to initiate the development of the fruit-body. The male gametic nucleus is delivered into the ascogonium, which differentiates into the hymenium. Karyogamy takes place in the hymenium and is immediately followed by meiosis. Subsequently, the haploid nuclei are packed into ascospores. Ultimately, the mature ascospores are forcibly discharged from fruit-bodies (perithecia).

**Figure 4:**
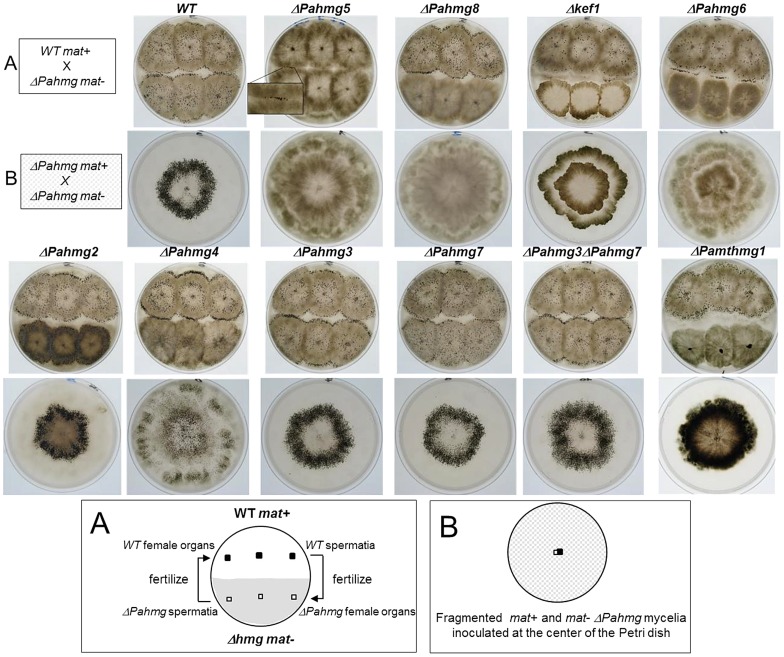
Crosses of HMG-box gene deletion mutants. (A) Analysis of male and female fertility of HMG-box gene deletion mutants in crosses with wild-type tester strains. When cultures were confluent, sterile water was poured and dispersed over the surface of the mycelium. As each strain produced spermatia (male cells) and protoperithecia (female organs) regardless of its mating type, reciprocal fertilization of the mutant and wild-type strains took place and indicated whether the mutant was fertile as a male (donor) or as a female (receptor). For *ΔPahmg5* some perithecia differentiated at the contact zone where mutant and wild-type mycelia fuse. In the resulting heterokaryotic mycelium, wild-type nuclei complement the male and/or female sterility defect of the mutant. Those perithecia were fertile and expelled numerous asci allowing genetic analysis. (B) Analysis of perithecium distribution in homozygous crosses of HMG-box gene deletion mutants. Fragmented mycelium from *mat+* and *mat−* strains with the same HMG-box deletion were deposited at the center of a Petri dish and incubated until perithecia formed. Typically, the wild-type strains differentiated perithecia within a ring-like area.

To determine whether HMG-box gene deletions impair sexual reproduction, each mutant strain was tested for male and female fertility in both mating types (*mat+* and *mat−*). The test for male fertility consisted in fertilizing wild-type female organs with spermatia from each HMG-box deletion mutant (Figure 4A). This analysis indicated that all mutants, except *ΔPahmg5*, produced functional spermatia. Moreover, fertilized female organs differentiated into mature fruit-bodies producing ascospores, indicating that HMG-box deletions in the male nuclei did not affect any developmental steps in the fertilized female organs. For each mutant, reciprocal crosses with *mat−* and *mat+* wild-type strains behaved similarly, indicating that the observed phenotype was not dependent on mating type. We further quantified spermatia produced by each deletion mutant and measured their activity in fertilization assays (Materials and Methods). Two mutants, *ΔPahmg4* and *Δkef1* produced five and seven times more spermatia, respectively, than the wild-type strain. Spermatia from these two mutants and from other mutants displayed the same fertilization ability as wild-type spermatia. The overproduction of spermatia observed in *Δkef1* was in agreement with microscopic observations, illustrating that even young hyphae produce spermatia. The *ΔPahmg5 mat+* and *ΔPahmg5 mat−* strains were found to produce as many spermatia as wild-type strains. Thus, male sterility in these *ΔPahmg5* strains can be attributed either to an inability of spermatia to fertilize wild-type protoperithecia or to an arrest of perithecial development shortly after fertilization.

To test the female fertility, we examined the formation of fruit-bodies by mycelia from each mutant fertilized with wild-type spermatia (Figure 4A). No fruit-bodies were produced on mutant mycelium in crosses involving *ΔPahmg5*, *ΔPahmg6*, *ΔPahmg8*, *Δkef1* and *Δmthmg1* strains, demonstrating that these mutants were sterile as female partners. In contrast, *ΔPahmg2*, *ΔPahmg3*, *ΔPahmg4* and *ΔPahmg7* strains produced fruit-bodies that developed normally and ejected as many ascospores as fruit-bodies from a wild-type cross. For each mutant, reciprocal crosses with *mat−* and *mat+* wild-type strains behaved similarly, indicating that the observed phenotype was not dependent on mating type.

We also examined the distribution of perithecia on the mycelium. These assays were based on homozygous crosses and were performed by inoculating a mixture of fragmented *mat+* and *mat−* mycelia in the center of a plate containing minimum medium under constant light illumination (Figure 4B). In this test, a wild-type strain differentiates fruit-bodies mostly in a ring-like area, which was 1 cm wide and located approximately 1 cm away from the inoculation point [70]. Only *ΔPahmg2* and *ΔPahmg7* differentiated fruit-bodies with the same pattern as the wild-type strain. The alterations were striking for *ΔPahmg4*, since fruit-bodies were distributed on the entire surface of the culture and the ring-like area was no longer visible. The pattern was also different for *ΔPahmg3*, which developed a wider ring-like fruit-body area. Furthermore, we observed that this phenotype was exacerbated by the deletion of *PaHMG7*. This difference was confirmed by quantitative measurement of the ring-like diameter in the *ΔPahmg3 ΔPahmg7* double mutant (Table 2). The increase in ring diameter (54.9 mm instead of 53.7 mm) observed in the double mutant suggested that the *PaHMG3* and *PaHMG7* genes together control the distribution of fruit-bodies. Fruit-bodies were never observed for *ΔPahmg5*, *ΔPahmg8* and *Δkef1*. The *ΔPahmg6* mutant did not produce fruit-bodies as fast as the wild-type strains, but extended incubation revealed that it was weakly female fertile. Fruit-bodies were 50 times less abundant than in a wild-type cross and they displayed an altered shape, with a smaller body and a larger neck than the wild-type fruit-bodies (data not shown). They began to eject ascospores 8 days after fertilization, whereas completion of the sexual process took 4 days in a wild-type cross. Genetic analysis of their progeny confirmed that they were produced by *ΔPahmg6* parents and not by contaminating mycelium.

In conclusion, all the eight HMG-box deletion strains analyzed in this study are altered phenotypically. Complementation assays confirmed that the deletion was responsible for the observed phenotype (Materials and Methods). None of the eight HMG-box genes analyzed in this study were essential for viability, although the deletion of three genes (*PaHMG2*, *PaHMG6 and KEF1*) resulted in growth alterations. Most HMG-box gene deletions affected sexual reproduction (Table 2). *PaHMG2* is the only *P. anserina* HMG-box gene whose deletion exclusively affected vegetative growth. Deletions of *PaHMG3*, *PaHMG4* and *PaHMG7* affected the distribution of fruit-bodies. More importantly, these deletions failed to affect vegetative growth and fruit-body development. Deletion of *PaHMG6*, *PaHMG8* and *KEF1* impaired female fertility, but mutant strains remained male fertile. Strikingly, the *ΔPahmg5* mutant exhibits both male and female sterility, an uncommon phenotype in *P. anserina* that was only reported for mating-type mutants [26].

### Heterokaryotic complementation of female sterility caused by HMG-box gene deletions

Microscopic observation of *ΔPahmg5*, *ΔPahmg6*, *ΔPahmg8* and *Δkef1* cultures revealed the presence of protoperithecia, indicating that female sterility was not due to the absence of female reproductive structures. Female sterility of *ΔPahmg5*, *ΔPahmg6*, *ΔPahmg8* and *Δkef1* can be attributed to a defect either in the development of the fruit-body envelope or in the formation of the hymenium, or both. To determine which tissue requires the HMG-box proteins, we performed trikaryon mosaics. In this experiment, *mat+* and *mat−* HMG-box mutant strains were co-cultured with a strain containing a deletion of the *mat+* idiomorph (*Δmat*) [71]. Except for the loss of the *mat+* mating-type gene, the *Δmat* strain had a wild-type genotype. Because the *Δmat* strain lacked the mating-type gene needed for fertilization, it could not participate in sexual reproduction and, thus, could not rescue hymenium defects. However, the *Δmat* strain maintained wild-type vegetative characteristics and could act as a helper strain by complementing maternal defects in a contest of sexually compatible mutant strains. Notably, the *Δmat* strain can provide maternal haploid tissues to form the perithecial envelope [72].

Fertile fruit-bodies were observed in the three trikaryotic cultures of *ΔPahmg6*, *ΔPahmg8* and *Δkef1* (data not shown), indicating that *Δmat* cells surrounding the hymenia of these HMG-box mutants promote the development of functional perithecia. These results demonstrated that PaHMG6, PaHMG8 and KEF1 are required for making the perithecial envelope and that they are dispensable for the hymenium. We also examined the behaviour of the *Δmthmg1* strain in a trikaryon mosaic test. As previously described [44], most *Δmthmg1* cultures died quickly and did not differentiate protoperithecia. A trikaryotic culture involving sexually compatible *Δmthmg1* strains and *Δmat* strain produced perithecia, but no ascospores were formed (data not shown). These results indicate that the *Δmat* tissue complemented the vegetative requirement for mtHMG1 during protoperithecium formation, while the absence of mtHMG1 in the hymenium led to an arrest of its development. The protein mtHMG1 is therefore required for both protoperithecial and hymenium development.

Trikaryotic cultures of *ΔPahmg5* did not produce any perithecia. It is noteworthy that mating-type gene mutations do not affect *PaHMG5* transcription [73], thus excluding the possibility that the *Δmat* strain is unable to complement the *ΔPahmg5* defects because it is itself affected for *PaHMG5* expression. Failure to restore fertility of *ΔPahmg5* in the trikaryotic test could reflect the need for this gene in both male and female fertility, which can be difficult to be restored simultaneously. Therefore, we designed an assay to separately evaluate the restoration of female and male fertility of *ΔPahmg5*. To evaluate whether the female sterility of *ΔPahmg5* could be rescued by a *Δmat PaHMG5^+^* strain, co-cultures of *mat+ ΔPahmg5* and *Δmat PaHMG5^+^* strains were used as female partners in crosses fertilized by *mat−* wild-type spermatia. Conversely, to evaluate whether *Δmat PaHMG5^+^* could restore the male competency of *ΔPahmg5*, cultures of the *mat−* wild-type strain were fertilized with spermatia issued from a co-culture of *mat+ ΔPahmg5* and *Δmat PaHMG5^+^* strains (Materials and Methods). Two fruit-bodies were observed on 10 Petri dishes for the female sterility restoration assay, while three fruit-bodies were observed on 10 *mat−* plates for the male restoration assay. Several thousand perithecia were formed with wild-type strains in similar experiments, indicating that male and female sterility of *ΔPahmg5* strain was inefficiently complemented by the *Δmat PaHMG5^+^* strain. Taken together, these data are consistent with a requirement of PaHMG5 for fertilization or an early stage of hymenium development. The arrest in the development of the hymenium at an early stage precludes any conclusion on the role of PaHMG5 in the development of the maternal perithecial envelope.

### Defects caused by overexpression of *PaHMG5*

In the complementation assay of the *ΔPahmg5* mutant, introduction of the *PaHMG5* wild-type allele restored male fertility but transformants remained female sterile (Materials and Methods). Moreover, the transformants displaying the highest efficiency as male partners were vegetatively altered, displaying a flat vegetative mycelium without aerial hyphae. These data suggested that deregulation of *PaHMG5* may be detrimental to the fungus. To examine the consequences of unregulated PaHMG5 expression, a plasmid was constructed to express a fusion of *PaHMG5* with the *Pagpd* (glyceraldehyde-3 phosphate-dehydrogenase) promoter and initiation codon [74] (Materials and Methods). When this fusion construct was introduced into protoplasts from the wild-type strain, most of the recovered transformants displayed a flat mycelium. In addition, they exhibited female sterility in crosses with the wild-type strain. Genetic analysis (Materials and Methods) demonstrated that the vegetative defect (flat mycelium) and female sterility consistently co-segregated with the *Pagpd::PaHMG5* transgene. These observations strongly suggested that female fertility relies on tight regulation of *PaHMG5* expression.

### Transcriptional expression of mating-related HMG-box genes and downstream target genes

**Figure 5:**
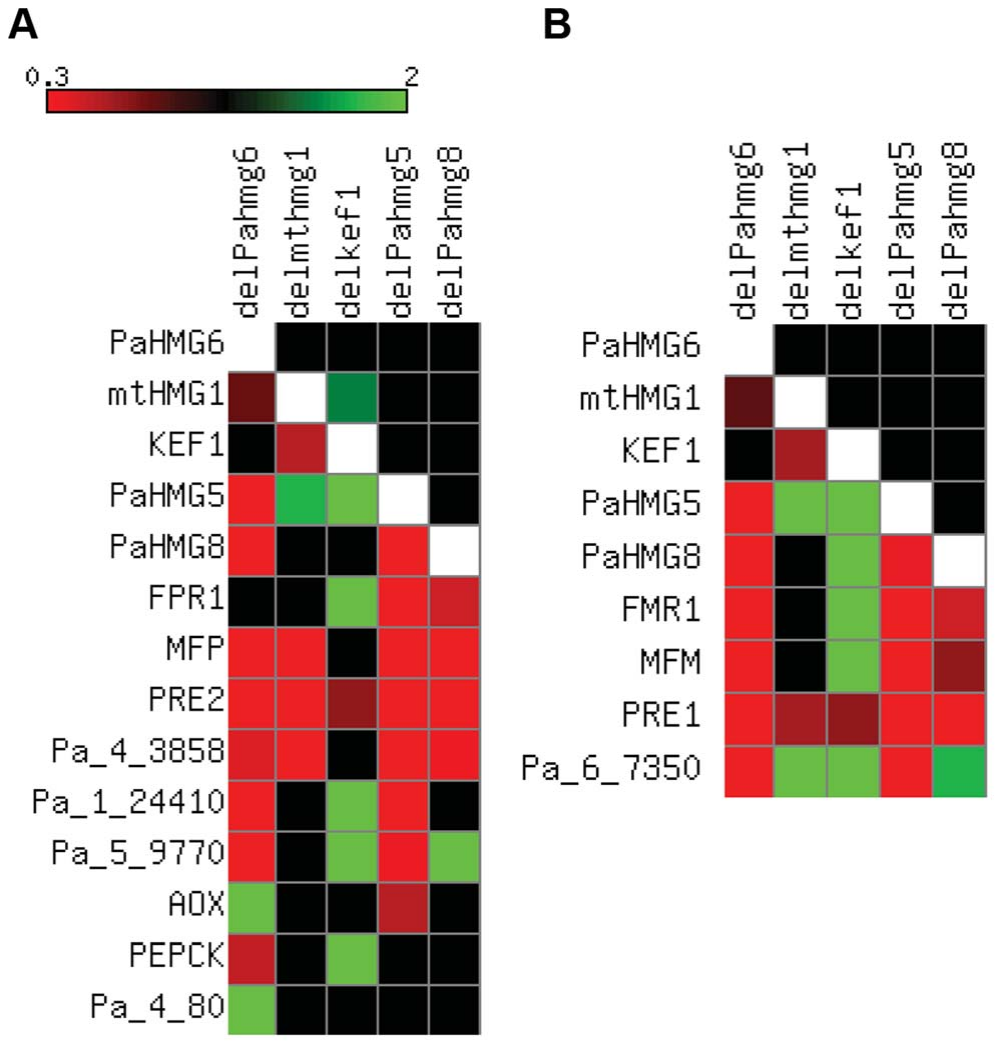
Expression of HMG-box genes and mating-type target genes in strains with HMG-box gene deletion. FCs from Table S3 to S7 were converted to a heat map using Matrix2png [108]. Squares for FCs with non-significant statistical values are in black. Squares for inapplicable values are in white. (A) Heat map in the *mat+* strains. The FCs of HMG-box genes, *FPR1* and selected FPR1 target genes are represented as indicated on the scale for the following strains: *mat+ ΔPahmg6* (delPahmg6), *mat+ Δmthmg1* (delmthmg1), *mat+ Δkef1* (delkef1), *mat+ ΔPahmg5* (delPahmg5), and *mat+ ΔPahmg8* (delPahmg8). Gene number: *MFP*, Pa_2_2310; *PRE2*, Pa_4_1380; *AOX*, Pa_3_1710; *PEPCK*, Pa_4_3160. (B) Heat map in the *mat−* strains. The FCs of HMG-box genes, *FMR1* and selected FMR1 target genes were represented as indicated on the scale in (A) for the following strains: *mat− ΔPahmg6* (delPahmg6), *mat− Δmthmg1* (delmthmg1), *mat− Δkef1* (delkef1), *mat− ΔPahmg5* (delPahmg5), and *mat− ΔPahmg8* (delPahmg8). Gene number: *MFM*, Pa_1_8290; *PRE1*, Pa_7_9070.

**Figure 6:**
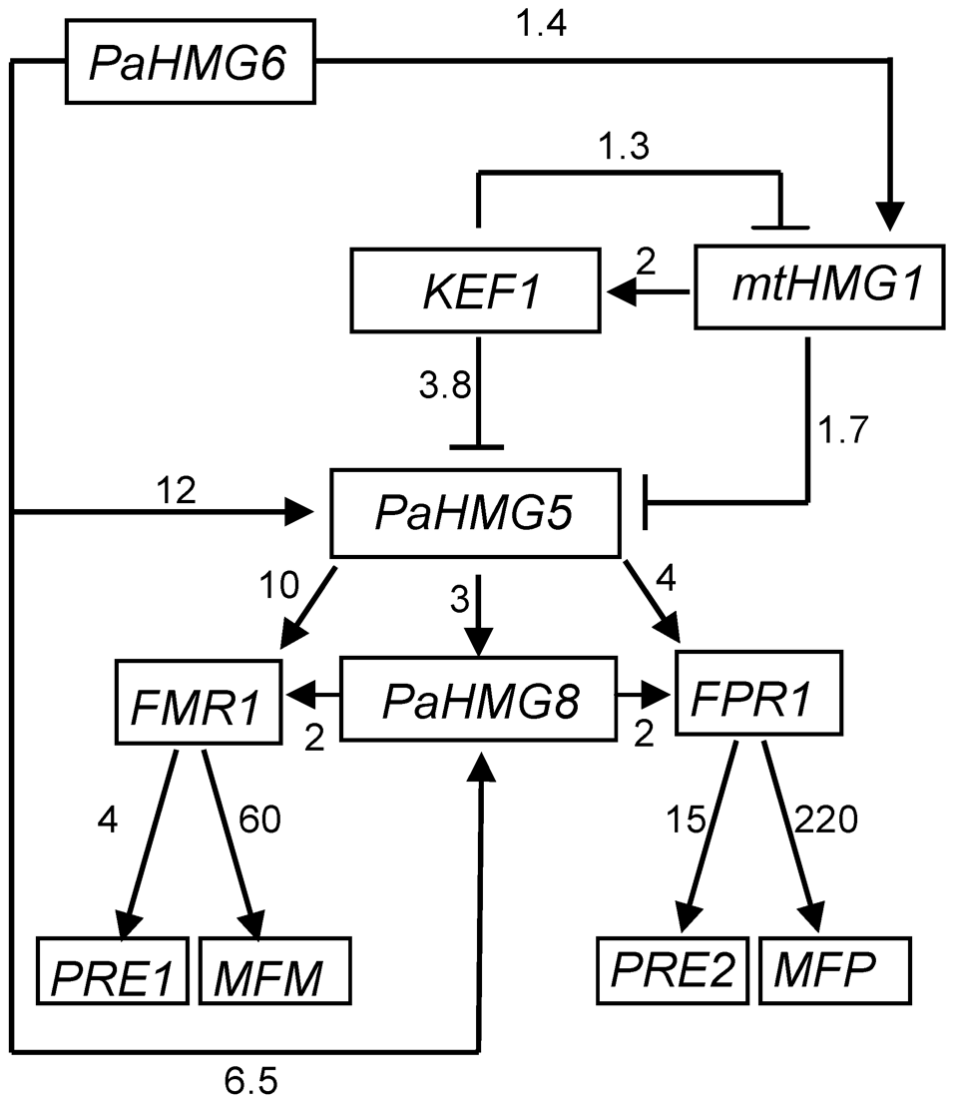
Genetic network of HMG-box genes that regulate mating in *P. anserina*. Arrows with heads and blunt ends indicate activation and repression, respectively. The numbers next to the arrows indicate the average FC in gene expression between the wild-type and mutant strains.The relationship between *mtHMG1* and *PaHMG5* may be mediated by *KEF1*. Alternatively, *mtHMG1* may by-pass *KEF1* to repress *PaHMG5* directly or indirectly. The consistency in the FCs suggest that *PaHMG6* by-passes this cascade to activate *PaHMG5* and *PaHMG8* either directly or indirectly. The numbers next to the arrows connecting the mating-type genes (*FMR1* and *FPR1*) and the downstream target genes are FCs that were obtained from [73].

*PaHMG5*, *PaHMG6*, *PaHMG8*, *KEF1* and *mtHMG1* [44] are involved in the development of male and female organs, raising the question of their genetic interactions. To assess the relationships between these genes, quantitative real-time RT-PCR (RT-qPCR) was used to examine their expression patterns in *ΔPahmg5* (Table S3), *ΔPahmg6* (Table S4), *ΔPahmg8* (Table S5), *Δkef1* (Table S6) and *Δmthmg1* (Table S7) strains in a *mat+* and *mat−* context. Genes were defined as up-regulated in the mutant strain if the fold change (FC) was >1, with a p-value of <0.05 (see Materials and Methods for FC computation). On the other hand, genes were defined as downregulated in the mutant strain if 0<FC<1 with a p-value of <0.05. FCs with a 95% confidence interval including the value of 1 were not considered significant [75]. The results are summarized in Figure 5. Most of the deletions had similar effects in *mat+* and *mat−* strains. The few exceptions were statistically non-significant results in *mat+* or *mat−* strains, *e*.*g*., *mtHMG1* had a significant FC (1.35) in *mat+ Δkef1* relative to wild-type and a non-significant FC (1.1) in *mat− Δkef1* (Table S6). A map of the genetic interactions among *PaHMG5*, *PaHMG6*, *PaHMG8*, *KEF1* and *mtHMG1* was constructed based on the assumption that deletion of a regulatory gene can affect downstream target genes, while non–affected genes are either upstream regulatory genes or genes acting in an independent pathway (Figure 6). Interestingly, *PaHMG6*, *PaHMG8*, *KEF1* and *mtHMG1* appeared to interact and converge on a key regulator, *PaHMG5*. We detected an effect of mtHMG1 on the transcription of *KEF1* and downstream HMG-box genes, suggesting that this protein is localized to the nucleus although it has been shown to be targeted to the mitochondria [44]. Re-examination of its sequence using PSORTII [76] revealed the presence of several monopartite and bipartite nuclear localization signals.

We further examined the transcription levels of mating-type genes and of a selection of specific target genes [73], including the pheromone and pheromone receptor genes, in the different mutant strains (Table S3 to S7 and Figure 5). We found that the transcription of the mating-type genes *FMR1* and *FPR1* was reduced in *ΔPahmg5* and *ΔPahmg8* strains. As expected from the cascade shown in Figure 6, *FMR1* was downregulated in *mat− ΔPahmg6*; in contrast, we failed to identify a significant reduction in *FPR1* transcription in *mat+ ΔPahmg6*. However, all tested FPR1 target genes were either up- or downregulated in this latter strain, suggesting that PaHMG6 by-passes FPR1 to control its target genes. Overall, transcription of the mating-type target genes increased in *Δkef1* strains, in agreement with the repressor effect of KEF1 on mating-type genes. Interestingly, transcription of pheromone receptor genes decreased in female sterile HMG-box mutants, even in the *Δkef1* strain. This is in agreement with their essential role in female fertility [73].

PaHMG5 appears to be the major regulator of *FPR1* and *FMR1* mating-type genes in the network presented in Figure 6. This raises the possibility that constitutive expression of *FPR1* and *FMR1* could compensate for the absence of PaHMG5 and, thus, rescue sterility of the *ΔPahmg5* mutant. Transgenic versions of *FMR1* and *FPR1* driven by the *Angpd* constitutive promoter were previously reported to complement loss-of-function of the corresponding gene [77]. Functional *Angpd-FMR1* or *Angpd-FPR1* transgenes were introduced by genetic crosses in *mat−* and *mat+ ΔPahmg5* mutant strains, respectively. We observed that *ΔPahmg5* strains carrying these transgenes remained male and female sterile, indicating that PaHMG5 function is not limited to the transcriptional activation of mating-type genes. It may regulate mating-type target genes as a cofactor of mating-type transcription factors. Alternatively, it may regulate fertility genes that are different from mating-type target genes.

### Promoter region analysis of HMG-box genes and target genes of HMG-box transcription factors reveals HMG-box binding sites

**Figure 7:**
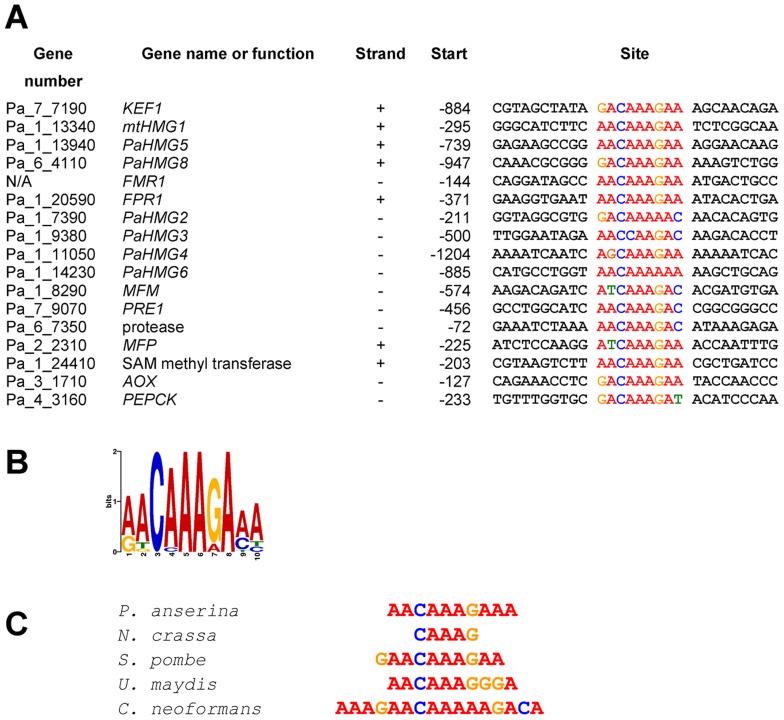
Conserved sequences found in the promoter region of HMG-box genes and mating-type target genes. (A) Conserved sequences from a number of HMG-box genes and mating-type target genes identified using MEME program [78]. Position −1 is the first nucleotide upstream of the translation initiation codon. (B) Weblogo of the consensus sequence generated by MEME (assembled from sequences listed in A). (C) The *P. anserina* consensus sequence aligned with the binding site of HMG-box proteins: MATa-1 (*N. crassa*) [109], SpSte11 (*S. pombe*) [30], Prf2 (*U. maydis*) [38], [39] and Mat2 (*C. neoformans*) [41].

The regulatory network shown in Figure 6 consists of a cascade of HMG-box genes, suggesting that each gene may contain a binding site for the upstream regulating HMG-box factor. A search for a conserved binding site using MEME [78] identified a consensus motif (A/G)ACAAAGAA in *KEF1*, *mtHMG1*, *PaHMG5*, *PaHMG8*, and the *FMR1* and *FPR1* mating-type genes (Figure 7A). This consensus motif is very similar to the common core DNA motif A(A/T)CAA(A/T)G that is recognized by HMG-box transcription factors [79] (reviewed in [2]). The remaining *P. anserina* HMG-box genes either contained a sequence that displayed some differences to the A(A/T)CAA(A/T)G core sequence (*PaHMG2*, *PaHMG3*, *PaHMG4* and *PaHMG6*) (Figure 7A), or they did not contain any related sequence (*PaHMG7* and *SMR2*). Further analyses of mating-type target genes using MEME revealed that the *mat+* pheromone receptor gene (*PRE1*), alternative oxydase gene (*AOX*), phospho-enol pyruvate kinase gene (*PEPCK*), *Pa_1_24410* and *Pa_6_7350* also contained the core HMG-box binding site ACAAAGA (Figure 7A). Interestingly, the two pheromone genes (*MFM* and *MFP*) displayed the same conserved core sequence, ATCAAAG. The *mat−* pheromone receptor (*PRE2*), *Pa_4_80*, *Pa_4_3858* and *Pa_5_9770* did not contain the core HMG-box binding site, suggesting that these genes are secondary targets of HMG-box genes. A total of eight genes contained the (A/G)ACAAAGAA consensus site. The comparison with the distribution of this site in the *P*. *anserina* genome indicated that the consensus site is significantly enriched in the selected set of genes examined here (p-value = 0.016) (Materials and Methods).

### Electrophoretic mobility shift assays

**Figure 8:**
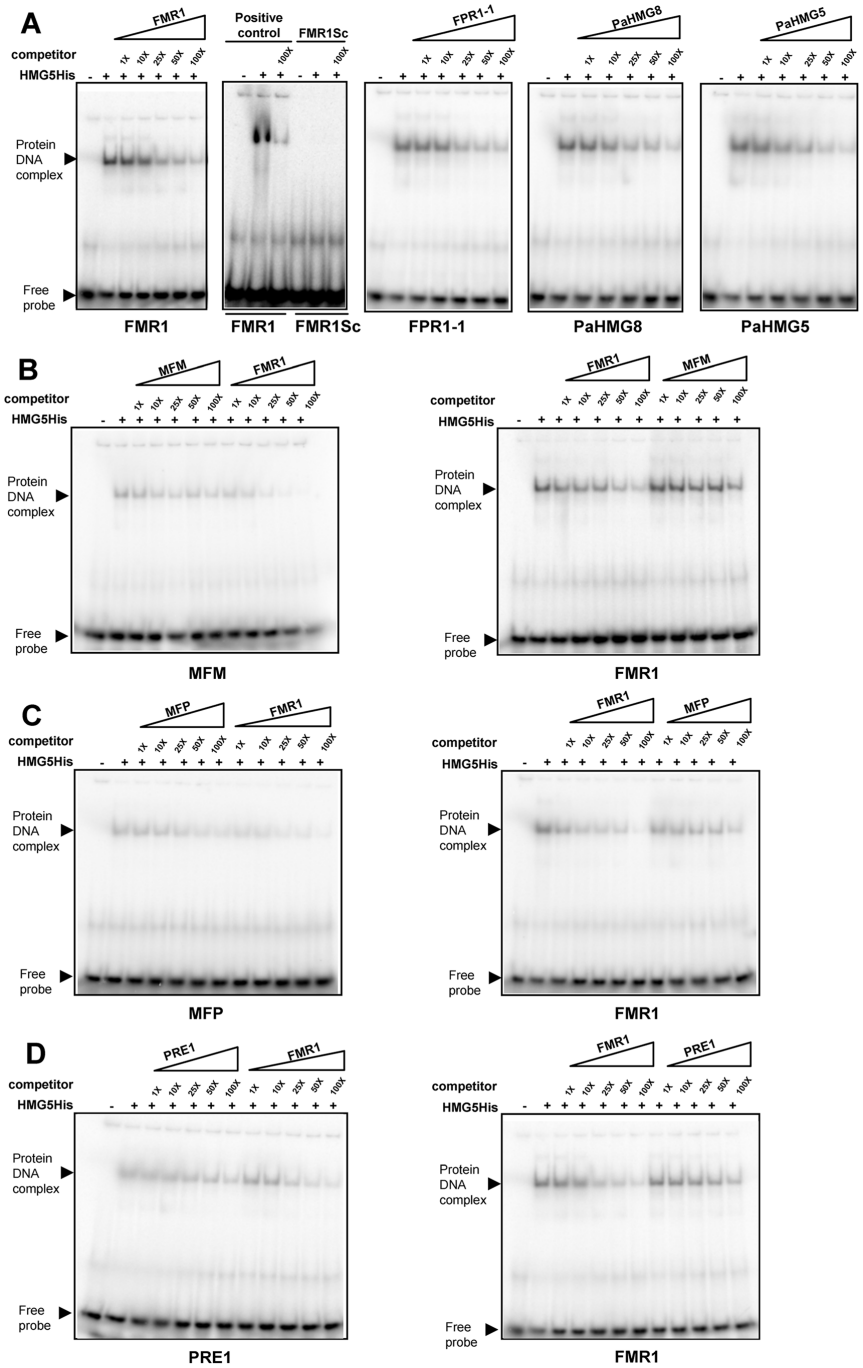
Electrophoretic mobility shift assays with PaHMG5. (A) Interaction of His tagged PaHMG5 (HMG5His) with probes corresponding to FMR1, FMR1 scrambled sequence (FMR1Sc), FPR1, PaHMG8 and PaHMG5 oligonucleotides. The probes are indicated below each panel. The interaction of HMG5His with probe was analyzed without competitor and in the presence of increasing amounts (given as fold molar excess below the triangles) of competitor (indicated above the triangles). (B) Interaction of the HMG5His protein with the MFM probe and reciprocal competition between FMR1 and MFM oligonucleotides. The probes are indicated below each panel. The competitors are indicated above the triangles. A control of the interaction of HMG5His with the FMR1 probe was performed as in A and included in the assay. HMG5His has a greater affinity for FMR1 than for MFM sequence, as indicated by the efficient exclusion of MFM probe by FMR1 competitor and the very inefficient exclusion of FMR1 probe by MFM competitor. (C) Interaction of the HMG5His protein with the MFP probe and reciprocal competition between FMR1 and MFP oligonucleotides. Legend as in B. HMG5His has a greater affinity for FMR1 than for MFP sequence, as indicated by the efficient exclusion of MFP probe by FMR1 competitor and the inefficient exclusion of FMR1 probe by MFP competitor. (D) Interaction of the HMG5His protein with the PRE1 probe and reciprocal competition between FMR1 and PRE1 oligonucleotides. Legend as in B. HMG5His has a greater affinity for FMR1 than for PRE1 oligonucleotides, as indicated by the efficient exclusion of PRE1 probe by FMR1 competitor and the inefficient exclusion of FMR1 probe by PRE1 competitor.

We further tested the binding of the heterologously expressed His-tagged version of the entire PaHMG5 protein (HMG5His) in electrophoretic mobility shift assays (EMSAs) with primers containing the putative binding sites identified above. We first examined the binding of HMG5His to its putative target genes (*FMR1*, *FPR1* and *PaHMG8*, Figure 6), including *PaHMG5*. These genes contained the consensus site (A/G)ACAAAGAA. Under the binding conditions used, HMG5His strongly bound to primers containing these sequences and showed decreased binding upon addition of unlabelled competitor (Figure 8A). Moreover, a primer with a scrambled *FMR1* sequence did not display any binding of HMG5His, confirming the specificity of the sequence recognition. These experiments support the idea that PaHMG5 directly regulates these target genes as well as its own transcription, as previously demonstrated for SpSte11 [80]. We further examined the affinity of HMG5His for the putative binding sites of the mating-type target genes (Figure S3), which displayed binding sites different from the consensus. Binding of HMG5His to sites that were different from the consensus (A/G)ACAAAGAA was much weaker than to the consensus binding site (see MFM, MFP AOX and PEPCK in Figure S3). The binding to *MFM*, *MFP* and *PRE1* sites was more carefully investigated (Figure 8, B to D). Reciprocal competition of *MFM*, *MFP* and *PRE1* sites with the *FMR1* consensus site confirmed that HMG5His had a greater affinity for the consensus sequence than for the sites of these mating-type target genes. These results indicate that PaHMG5 recognized the sites in the 5′UTR of mating-type target genes, but may require the mating-type transcription factors to increase the efficiency of binding. We also evaluated the affinity of HMG5His for the putative binding sites of the HMG-box genes that are not regulated by PaHMG5 in our proposed network (Figure 6). HMG5His bound strongly to *KEF1* and *mtHMG1* sites, which are identical to the consensus sequence, while binding to *PaHMG2*, *PaHMG3*, *PaHMG4* and *PaHMG6* sites was much less efficient (Figure S4). Strikingly, the less efficient binding occurred with sites that were different from the consensus by modification of the central part of the site (see PaHMG2, PahMG*3* and PaHMG6 in Figure S4). Reciprocal competition confirmed that HMG5His has much less affinity for the *PaHMG2*, *PaHMG3*, *PaHMG4* and *PaHMG6* sites than for the consensus site (Figure S5). We assume that the binding of HMG5His to these sites corresponded to the recognition of an HMG-box binding site by an HMG-box protein, but this did not demonstrate *in vivo* regulation by PaHMG5.

The Weblogo (Figure 7B) showed a consensus binding site obtained from the entire set of analyzed sequences. This consensus corresponded to the sequence that was recognized with the best efficiency by HMG5His in EMSA. Comparison of this consensus with other HMG-box binding sites (Figure 7C) reveals a striking similarity with the TR-box, which is bound by SpSte11 of *S*. *pombe* [30], [81]. The *P*. *anserina* consensus site matched the TR-box in nine consecutive basepairs. The similarity of the HMG5His consensus binding site with the PRE-boxes from *U. maydis* and *C. neoformans* was reduced. In particular, the PRE-box of *U*. *maydis* had an A instead of a G in position 7 in the *P*. *anserina* consensus. This transition correlates with a weak binding of HMG5His (see PaHMG2 and PaHMG6 in Figure S4). Taken together, these data indicate that PaHMG5 binds most efficiently to sites that are almost identical to those that are recognized by SpSte11, its ortholog in *S. pombe*.

An analysis of the distribution of the (A/G)ACAAAGAA binding site in the *P. anserina* genome indicated that this motif is preferentially localized on segments of 1000 bp upstream of the predicted translational start sites (p-value of <0.0001) (Materials and Methods). In contrast, a scrambled binding site did not display significant enrichment in these 1000 bp regions corresponding to promoter regions (p-value = 0.08). A total of 502 genes contain the (A/G)ACAAAGAA motif, suggesting that up to 5% of *P. anserina* genes may be directly controled by MATA_HMG-box transcription factors.

## Discussion

### Significance and conservation of the HMG-box gene functions in fungi

The data presented here provide new insights into the role and the relationships of HMG-box genes in the fungus *P. anserina* (Pezizomycotina). We revealed a network of HMG-box genes upstream of the *FMR1* and *FPR1* mating-type genes, which are themselves HMG-box genes. PaHMG5 plays a central role in this network by regulating mating-type gene transcription. We identified SpSte11 as the ortholog of PaHMG5 in *S*. *pombe* by different phylogenetic analyses, and we demonstrated that both recognize almost identical binding sites. KEF1 is another important member of the network, acting upstream of *PaHMG5* as a repressor. KEF1 also appears as a repressor of hyphal anastomoses, which are dramatically deregulated in a *Δkef1* strain. Moreover, the control of mating-type target genes by FMR1 and FPR1 was described in a previous report [73], which, together with the results presented here, provides the first exhaustive view of the regulatory circuits upstream and downstream of mating-type genes in a filamentous Ascomycete.

Strikingly, ten of the 12 HMG-box genes identified in the genome of *P. anserina* control fertility and sexual development. Although two genes (*PaHMG2* and *PaHMG7*) were not directly involved in sexual reproduction, the deletion of *PaHMG7* displayed a synergistic effect with the deletion of *PaHMG3* on the perithecium distribution in co-cultures of *mat−* and *mat+* strains. This observation demonstrates that 11 of the HMG-box genes have a direct or indirect function during sexual reproduction. The genome-wide systematic deletion analysis of *F. graminearum* transcription factors [43] allowed us to compare the resulting phenotypes with those of *P. anserina* (Table S8). A total of six out of the 11 HMG-box genes deleted from *F*. *graminearum* were involved in perithecium development. One HMG-box gene (*FGSG_06760*) was not deleted, and the perithecium distribution on the mycelium was not tested in *F. graminearum* as described here. Taken together, these data implicate the HMGB superfamily in sexual reproduction in Pezizomycotina, although further analyses in other species should validate this finding.

Comparative analyses of sex regulatory pathways indicate that transcription factors have often evolved to accommodate unique rather than conserved functions, even across closely related lineages [82], [83] (reviewed in [84], [85]). For example, the pheromone-response pathway is controlled by an HMG-box protein in *C*. *neoformans* [41] and a transcription factor distantly related to the homeodomain family in *S*. *cerevisiae* [86], providing evidence for a dramatic change in a key regulator in these species, despite the presence of strong effector conservation [41]. Our study revealed that PaHMG5 and SpSte11 [30] are striking exceptions to the unusual plasticity of pathways regulating sex. These two orthologous HMG-box proteins positively regulate mating-type gene transcription in *P. anserina* and *S. pombe*. Moreover, the transcription factor analysis published by Son *et al* [43] suggests that the SpSte11 ortholog in *F*. *graminearum* (FGSG_01366, *GzHMG010*) also shares this conserved function. The phenotype of *F*. *graminearum* deleted for *GzHMG010* recapitulates the phenotype of mating-type gene deletions (Table S8, see *GzHMG010*, *MAT1-1-1* and *MAT1-2-1*) and mating-type locus deletion [87]. This feature is expected for genes operating in the same pathway, but molecular analyses will be necessary to determine whether the control of mating-type gene expression by SpSte11 orthologs extends to other fungi. *N. crassa* contains two co-orthologs of *PaHMG5*, indicating that control of mating-type gene expression in *N*. *crassa* may be more complex than in *P. anserina*. Based on phylogenetic analyses and analyses of synteny, we identified *NCU09387* as the ortholog of *PaHMG5*, while *NCU02326*, the inparalog of *NCU09387*, has no counterpart in *P. anserina*. Mutations of *NCU09387* resulted in strains that could mate, but fruit-body development arrested before ascospore formation [55], [56]. This phenotype is clearly different from the mating-type deletion, which resulted in a strain that is unable to mate [88]. This observation raises the interesting possibility that NCU02326 may control the expression of mating-type genes during fertilization, while NCU09387 is involved in the regulation of mating-type genes after fertilization.

### The HMG-box gene network regulates vegetative and mating-type fertility genes

Sexual reproduction relies on an interplay between vegetative tissues (the mycelium and the maternal hyphae) and sexual tissue (the hymenium). Vegetative tissues critically contribute to optimal conditions for ascogonium formation and to fertility by providing nutrients to growing fruit-bodies [68]. Inside the fruit-body, the hymenium goes through karyogamy and meiosis, and provides signals to the vegetative tissues to sustain nutrient mobilization [26]. This duality raises the question as to whether the alteration that entailed sterility in the mutant strains affects vegetative tissues, or sexual tissues via mating-type genes. The strains deleted for *PaHMG6*, *PaHMG8* and *KEF1* showed female sterility which was restored by complementation with the *Δmat* strain in trikaryotic mosaic tests. Although PaHMG6, PaHMG8 and KEF1 control transcription of mating-type genes, the trikaryotic test revealed that they also control fertility genes that are independent of mating-type genes but, nevertheless, critical for sexual reproduction.

The regulatory circuit shown in Figure 6 and the complete sterility resulting from the *PaHMG5* deletion points to this gene being a major regulator of sexual reproduction in *mat+* and *mat−* strains. We demonstrated that this gene controls mating-type genes and the pheromone/receptor systems. Pheromone and pheromone receptor genes were identified as the most critical mating-type targets for male and female fertility, respectively [73], [89]. The deletion of *PaHMG5* had a moderate effect on the transcription of mating-type genes (*FPR1*, FC = 0.25; *FMR1*, FC = 0.1; Table S3), but strongly reduced the transcription of pheromone genes (*MFP*, FC = 0.0004; *MFM*, FC = 0.004; Table S3). This effect on pheromone genes can explain the male sterility phenotype of *ΔPahmg5* strains. Moreover, some genes necessary for the biogenesis of pheromones are regulated by mating-type genes [73], and their transcription may also be decreased in *ΔPahmg5* strains, thereby enhancing male sterility. By contrast, transcription of the *PRE2* receptor gene was reduced 4-fold, a value that is unlikely to result in complete female sterility, which was characteristic of the *mat+ ΔPahmg5* strain. This observation suggests that the deletion of *PaHMG5* affects the transcription of another target gene that is critical for female fertility. Further experiments will be necessary to identify this target gene and to determine if it is a target of mating-type genes or a vegetative critical fertility gene.

### Relationship between the stationary phase and the HMG-box gene network

Several lines of evidence indicate that the competence of *P*. *anserina* for sexual reproduction is acquired during the stationary phase. Development of reproductive structures takes place during this phase [90], and the expression of mating-type genes increases up to 1000-fold upon entry into the stationary phase [73]. Our study provides the first evidence of a link between the stationary phase and the HMG-box genes that control the sexual cycle. Deletion of *KEF1* resulted in the formation of anastomoses, appressorium-like structures, and spermatia in young hyphae, all hallmarks of the stationary phase. We propose that this gene is a critical repressor of the switch to the stationary phase, which underlies the morphological transitions of this stage. However, this gene only contributed moderately to the increase in mating-type gene expression, as its deletion resulted in a 3-fold increase of *FMR1* and *FPR1* transcription. Other pathways are likely to connect entry into the stationary phase and sexual competence. Two MAP kinase pathways, PaMpk2 and PaMpk1, have essential roles in establishing the stationary phase in *P. anserina* [67], [90], [91]. No experimental evidence is yet available in *P*. *anserina* to support the link between these MAP kinase pathways and HMG-box genes. Exhaustive analyses of MAP kinase pathways in *S*. *cerevisiae* and *S*. *pombe* revealed numerous connections with HMG-box proteins. For instance, SpSte11 is a direct target of the Spk1 MAP kinase pathway in *S*. *pombe* [92], suggesting that PaHMG5 may be a phosphorylation target of the Spk1 ortholog in *P. anserina* (PaMpk2). Another interesting connection was uncovered in *S*. *cerevisiae* between Nhp6p and the Mpk1p (Slk2p) MAP kinase pathway [61], which is orthologous to the PaMpk1 MAP kinase cascade in *P. anserina*. These connections provide future direction to find the molecular pathways linking the stationary phase and the sexual cycle.

### A module of two HMG-box genes involved in sexual development is present in animals and fungi

Sex determination is highly variable, in contrast to other developmental systems that are well conserved through evolution. Even within a single kingdom, studies on common laboratory model organisms reveal that the genetic mechanisms of sex determination bear little, if any, resemblance. Strikingly, PaHMG5 and SpSte11 have conserved their function as mating-type regulators in *P. anserina* and *S. pombe* [30], although these two organisms have diverged 550 million years ago [93]. Moreover, the mating-type genes in both fungi are themselves HMG-box-genes, thereby defining an HMG-box module that is conserved in both fungi. An HMG-box module involved in sexual reproduction is also present in the basidiomycete fungus *U. maydis*. The Rop1 protein directly regulates the transcription of *prf1*, another HMG-box gene [37]. The Prf1 protein in turn induces the expression of mating-type genes [38], [39]. HMG-box genes also play a critical role in sex determination in vertebrates. *Sry*, the mammalian Y-chromosomal testis-determining HMG-box gene is an activator of *Sox9*. *Sox9* is also conserved among non-mammalian vertebrate species and has an ancestral and pivotal role in sex determination [2]. The conservation of a similar regulatory HMG-box module in vertebrates and Dikarya reveals a commonality of sex regulation in animals and fungi. *Sry* and *Sox9* are not orthologs of the fungal HMGB module genes. The two modules are thus analogs, not homologs. However, Martin *et al* [27] noted that the SexM protein of *Phycomyces blakesleeanus* was classified within the SOX-TCF_HMG subfamily. This placement is confirmed in the phylogram presented here (Figure 2: SexM, Phybl8). The discovery of a SOX-TCF_HMG-box regulator of mating-type genes in *P*. *blakesleeanus* would indicate an ancestral origin for an HMG box module involved in sex determination in Opisthokonta. Further investigations on HMG-box transcription factors and sex regultation in fungi should provide relevant information about the conservation and evolution of such modules.

## Materials and Methods

### Strains and media

The genetic and biological features of *P*. *anserina* were first described by Rizet and Engelmann [94] and current culture techniques can be found at http://podospora.igmors.u-psud.fr/methods.php. The strains used in this study were all derived from the *S* strain [95], which was used to determine the *P*. *anserina* genome sequence [53].

### Identification of HMG-box genes and structural analyses

The *P. anserina* genome and protein databases are available at http://podospora.igmors.u-psud.fr/. Identification of HMG-box genes was checked using Fungal Transcription Factor (http://ftfd.snu.ac.kr/intro.php) [49] and the Superfamily databases (http://supfam.cs.bris.ac.uk/SUPERFAMILY/index.html) [50]. For domain identification, Pfam [96] and CD-searches [51] were run on http://pfam.sanger.ac.uk/ and http://www.ncbi.nlm.nih.gov/Structure/cdd/cdd.shtml, respectively. *N*. *crassa* HMG-box genes were obtained from http://www.broadinstitute.org/annotation/genome/neurospora/MultiHome.html. FUNGIpath [52] was used to identify systemically orthologous groups of genes (http://embg.igmors.u-psud.fr/fungipath/). Subcellular localization was predicted with PSORTII [76] at http://psort.hgc.jp/form2.html.

### Statistical test for HMG-box gene distribution in the *P*. *anserina* genome

A total of nine HMG-box genes mapped to chromosome I and three HMG-box genes mapped to other chromosomes. Among the nine genes that mapped to chromosome I, three mapped to the mating-type locus and corresponded to *mat+* and *mat−* idiomorphs (reviewed in [26]). The idiomorph considered for statistical analysis was the *mat*+ idiomorph, which contained one HMG-box gene (*FPR1*). The total number of HMG-box genes on chromosome I was therefore, seven. The sizes of chromosome I to VII were 8,813,526 bp, 5,165,605 bp, 4,712,833 bp, 3,808,397 bp 4,734,309 bp, 4,264,133 bp and 4,087,213 bp, respectively. The total size of the *P*. *anserina* genome was 35,686,016 bp. The expected number of HMG-box genes on chromosome I was between two and three. The p-value was calculated on a contingency table http://www.graphpad.com/quickcalcs/contingency1.cfm using Fisher's exact test (p-value = 0.07 for two genes; p-value = 0.18 for three genes). The expected number of HMG-box genes on chromosome I was not significantly different from seven genes.

### Phylogenetic analyses

Sequence acquisition, identification of consensus amino acids and phylogenetic analysis were performed as described previously [27]. The HMG-box domains used to build the phylogenetic tree (Figure 2) and their alignment are in dataset S1 and dataset S2, respectively.

### Gene deletion and complementation

To delete the chromosomal copy of the eight HMG-box genes, eight plasmids containing deletion cassettes conferring resistance to hygromycin B [Hyg^R^] were constructed according to the *N*. *crassa* strategy for high-throughput generation of gene deletion [97] with modifications aimed at minimizing errors in the 5′ and 3′ flanking regions [73] (see Table S9 for primer sequences). The deletion cassette was released from the vector by *Asc*I digestion prior to transformation of *ΔPaKu70* protoplasts [98]. This transformation assay consistently yielded a high percentage of transformants with the correct deletion (>90%). One or two transformants obtained from each assay were subjected to Southern blot analysis to confirm the deletion (Figure S2), and one transformant with the expected hybridization pattern was selected for further analysis. The eight primary transformants containing corresponding targeted deletions were genetically purified by crossing with a wild-type strain of opposite mating type. This eliminated untransformed nuclei and segregated out the *ΔPaKu70* mutation through its phleomycin resistant [Phleo^R^] phenotype. Screening for [Hyg^R^, Phleo^S^] strains allowed the identification of *mat+* and *mat−* strains containing the HMG-box deletion but lacking *ΔPaKu70*. These strains constituted the stock of the deletion mutant for subsequent studies.

To ensure that the phenotype(s) observed for the HMG-box gene deletion mutants was actually due to inactivation of the relevant gene, the wild-type allele was reintroduced by transformation into the corresponding mutant. Wild-type alleles were obtained by amplifying fragments encompassing the corresponding gene (see Table S10 for primer sequences) and these were used directly for co-transformation of the mutant strain with the pPable vector [77], which conferred resistance to phleomycin. A significant number of co-transformants displaying a restored wild-type phenotype were recovered in each assay, demonstrating that phenotypes were not due to additional mutations (Table S11). It should be noted that introduction of the *PaHMG5* wild-type allele into the Δ*PaHMG5* mutant only rescued the male defect without restoring female fertility, indicating partial complementation.

### Measure of spermatium production and activity

Mutants were grown for 7 and 14 days on Petri dishes containing minimal agar medium. Spermatia were recovered by washing the surface of the dish with 1.5 ml of water and were counted with a haemacytometer chamber. A diluted spermatium suspension was used to fertilize a wild-type strain of opposite mating type and perithecia were counted after incubation for 4 days. Typically, 30% to 50% of spermatia from the wild-type strain were fertilizing, giving rise to perithecia. Duplicates were carried out for all strains and the whole experiment was performed twice to confirm the data.

### Microscopy

Microscopic observations were made on 4-day-old mycelia growing on cellophane placed on solid M0 medium (minimal medium lacking dextrin as carbon source) in a Petri dish. Small pieces (1 cm^2^) of cellophane containing mycelium were cut with a scalpel and mounted upside down in water. Pictures were taken with a Leica DMIRE 2 microscope coupled to a 10 MHz Cool SNAPHQ charge-coupled device camera (Roper Instruments). Since penetration of the cellophane through apressorium-like structures occurs perpendicularly to the surface, pictures were obtained at one micrometer increments to capture this process. Stacks of pictures were analyzed with ImageJ (http://rsb.info.nih.gov/ij) and deconvolution was performed with CombineZP (Alan Bradlay; alan@micropics.org.uk). Calculations of the mean and the standard deviation of the distance between emergence of appressorium-like structures and the leading edge of the thallus were made using 20 and 10 individual measurements in wild-type and *ΔPahmg9* strains, respectively.

### Construction of the *ΔPahmg3 ΔPahmg7* double mutant

One heterokaryotic *ΔPahmg3 mat+*/*ΔPahmg7 mat−* culture was self-crossed, and *mat+* and *mat−* homokaryotic double mutants were isolated from the progeny. Single and double mutants displayed a [Hyg^R^] phenotype and were, thus, undistinguishable. Therefore, a search was performed for asci showing first division segregation of the [Hyg^R^] phenotype (*i.e*., asci with two [Hyg^R^] and two [Hyg^S^] ascospores). Sensitive ascospore-derived cultures from these asci carried wild-type alleles of both genes; hence, resistant ones harbored mutations in both.

### Deregulation of the *PaHMG5* gene

The plasmid pBHGSTE11 contains a fusion of the *P. anserina gpd* promoter and initiation codon [74] with the coding phase of *PaHMG5*. The *Pagpd* promoter was a 0.38 kbp fragment obtained from the pPable plasmid [77], digested with *Nco*I, treated with Klenow and digested with *Xba*I. The *PaHMG5* sequence was amplified with *Pfu* (Promega) from GA0AB103CF05 [53] using 5PSTE11 and 3HindSTE11 primers (see Table S10 for primer sequences) and digested with *Hind*III. The *Pagpd* promoter and the *PaHMG5* fragment were then ligated into the *Hind*III and *Xba*I sites of plasmid pBCHygro [99] to yield the pBHGSTE11 plasmid. Sequencing of the entire fusion confirmed that the first Pagpd codon was in frame with *PaHMG5* and that no mutation altered PaHMG5.The pBHGSTE11 plasmid was introduced into *mat+* wild-type protoplasts and 10 [Hyg^R^] transformants were phenotypically analyzed in a cross with a *mat−* wild-type strain. A total of nine transformants showed a flat and female sterile mycelium, but they were fertile as male partners. To determine more precisely the phenotypic effects resulting from the integration of the *Pagpd::PaHMG5* fusion, the progeny from three representative transformants were subjected to genetic analysis. Segregation of the *Pagpd::PaHMG5* fusion was scored through the [Hyg^R^] phenotype. Most unpigmented ascospores did not germinate. Their genotype could nevertheless be deduced from tetrad analysis. For two transformants, the presence of the *Pagpd::PaHMG5* fusion was responsible for an ascospore pigmentation and germination defect (although most unpigmented ascospores did not germinate their genotype could nevertheless be deduced from tetrad analysis). However, pigmented ascospores giving rise to a [Hyg^R^] mycelium were recovered in the same progeny. These displayed a similar phenotypic vegetative alteration as observed in the primary transformants (flat mycelium and female sterility). In a transformant corresponding to a different integration site (different second division segregation % of [Hyg^S^]/[Hyg^R^]), the presence of the *Pagpd::PaHMG5* fusion did not affect ascospore pigmentation; instead it conferred the vegetative mycelium defect (flat mycelium and female sterility). These data and conclusions were subsequently confirmed by analyzing second generation progeny which were obtained by crossing the purified [Hyg^R^] *Pagpd::PaHMG5* bearing transformants with the wild-type strain.

### RT-qPCR experiments

Vegetative cultures for RNA preparation were performed on Petri dishes containing minimal medium and covered with a cellophane sheet (Bio Rad Hercules, USA). These cultures were inoculated with nine implants from *mat+*, *mat−* or HMG-box mutants of either mating type. Dishes were placed at 27°C under constant light (0 h) and were removed from the incubation room at 96 h, at which time *P. anserina* was competent for fertilization [73]. Mycelia were harvested and RNAs were extracted as described previously [73]. Purified RNAs were submitted to an additional DNase digestion in solution and cleaned up once more on RNeasy Plant Mini Kit (Qiagen, Hilden, Germany). Total RNAs were reverse transcribed with SuperScript III (Lifes Technologies) according to manufacturer's instructions. Each time the expression of an intronless gene was quantified, a non-reverse transcribed (NRT) control was performed for each biological replicate. For genes with introns, all primers were designed against two consecutive exons (see Table S12 for primer sequences) and an NRT control was systematically performed on a pool of biological replicates. Each RT-qPCR experiment contained at least five biological replicates and each point was performed in technical duplicate. Normalization genes for *ΔPahmg5*, *ΔPahmg8* and *Δmthmg1* were selected from a pool of ten housekeeping genes using geNorm [100] as described previously [73]. geNorm failed to select normalization genes for *ΔPahmg6* and *ΔPahmg9* strains, probably because metabolism was altered in these strains. A single stable reference gene was identified in these strains using NormFinder [101]. The normalization genes are listed in Table S13. RT-qPCR normalization was performed according to the relative quantification method with kinetic PCR efficiency correction. Standard error and 95% confidence interval calculations, and other statistical analyses were performed using REST 2009 software (Qiagen, Hilden, Germany) [102]. The FC in the expression of a gene of interest was computed as the normalized relative quantity of cDNA in sample relative to that in the control:

FC = relative quantity of cDNA for the gene of interest x (geometric mean of relative quantity of cDNA for the normalization genes)^−1^

The relative quantity (RQ) of a cDNA was:

RQ = efficiency of amplification ^(arithmetic mean for WT strain replicates – arithmetic mean for mutant strain replicates)^

The efficiency of amplification was above 1.8 for all analyzed genes. Genes were defined as downregulated in the mutant strain if 0<FC<1 with a p-value of <0.05. On the other hand, genes were defined as up-regulated in the mutant strain if FC>1, with a p-value of <0.05. FCs with a 95% confidence interval including the value of 1 were not considered significant [75].

### Consensus motif search and analysis of HMG-box binding motif distribution

Motif searches were conducted using MEME http://meme.nbcr.net/meme/cgi-bin/meme.cgi [78] on segments of 1000 bp upstream of the predicted translational start sites. A group of core sequences including *KEF1*, *mtHMG1*, *PaHMG5*, *PaHMG8*, and the *FMR1* and *FPR1* mating-type genes was first analyzed using MEME to identify the consensus HMG-box binding site. Subsequently, each candidate sequence was included with the core sequences for MEME analysis. Segments of 1500 bp upstream of the predicted translational start sites were analyzed when genes yielded no hit using MEME. The number of occurrences of the (A/G)ACAAAGAA binding site was counted for the *P*. *anserina* genome (n = 1149 in 35,686,016 bp). Assuming a random distribution throughout the genome, the (A/G)ACAAAGAA sequence is expected to occur 0.55 time in the promoter regions of the genes selected for MEME analysis (17,000 bp). The observed and expected numbers of (A/G)ACAAAGAA sequence were compared on a contingency table (http://www.graphpad.com/quickcalcs/contingency1.cfm) and a p-value was computed using Fisher's exact test. The (A/G)ACAAAGAA sequence is significantly enriched in the selected set of gene analyzed with MEME (p-value = 0.016). We further analyzed the distribution of the (A/G)ACAAAGAA sequence in the promoter region of all *P. anserina* genes. The number of occurrences of the (A/G)ACAAAGAA sequene was counted on segments of 1000 bp upstream from the predicted translational start site of each *P*. *anserina* gene (n = 502 in 10,460,890 bp). Assuming a random distribution throughout the genome, the (A/G)ACAAAGAA sequence is expected to occur 339 times in the 10,460,890 bp of the promoter region DNA. Thus, the observed distribution of the [(G/A)ACAAAGAA] sequence in the promoter sequences is significantly different from the random distribution (p-value of <0.0001). The scrambled PaHMG5 binding site [A(G/A)AAGAACA] occurred 1398 times in the genome of *P. anserina* and 457 times in the promoter regions. A random distribution of the scrambled site would result in 413 occurrences in promoter regions. Thus, the observed distribution of the [(G/A)ACAAAGAA] sequence in promoter regions is not significantly different from the random distribution (p-value = 0.08).

### Protein expression, purification and electrophoretic mobility shift assays

The full length PaHMG5 cDNA was amplified by PCR with LA Taq (TaKaRa, Shiga, Japan) from reverse transcribed total RNAs with primers Nde13 and HisBam13 (Table S10) according to the manufacturer's instructions. HisBam13 was designed to introduce an His_6_ tag downstream of the 3′ coding sequence. The PCR products were cloned into the pET28 vector (Novagen) between *Nde*I and *Bam*HI restriction sites and inserts were sequenced to identify cDNA without mutations. *E. coli* BL21 (DE3) transformed with the recombinant PET28 vector was grown in 2×YT medium (MP Biomedicals) supplemented with kanamycin at 50 µg/ml. Approximately 800 ml of culture medium was incubated in a shaker at 200 rpm at 37°C until OD600∼0.6–0.8. Protein expression was then induced with 0.5 mM isopropyl β-D-thiogalactopyranoside (Sigma) and the cell culture was further incubated at 15°C overnight. Cells were harvested by centrifugation, resuspended in 40 ml of 20 mM Tris pH9.0, 500 mM NaCl, 5 mM β-mercaptoethanol and protease inhibitor cocktail (Roche), and stored at −20°C. Cell lysis was achieved by sonication, and the cell extract was centrifuged at 20000 *g* for 30 min at 4°C. The His-tagged protein from the soluble fraction was purified on a nickel-nitrilotriacetic acid column (Qiagen Inc.) and eluted with an isocratic imidazole gradient, followed by a cation exchange step on a HiTrap Heparin column (GE Healthcare) equilibrated in 20 mM Tris (pH9.0), 300 mM NaCl, 5 mM β-mercaptoethanol, and 5% glycerol. The protein was eluted using a linear salt gradient. The production of recombinant protein was confirmed by SDS-PAGE. Lysis, soluble and some purified fractions were tested by western blot analysis. Proteins were resolved by SDS-PAGE and transferred onto nitrocellulose membrane (Protran, Whatman). The membrane was blocked by incubation for 1 hour at room temperature with 5% nonfat milk in TBS-T (Tris buffered saline-Tween 20, pH 7.5), incubated with rocking for 1 hour with anti-6His IgG conjugated with peroxidase (1/2,000) (Roche), and developed using the BM Blue POD substrate reagents from Roche.

Complementary primers (Table S14) were annealed to yield double stranded 33 bp oligonucleotides with single base 5′ overhangs consisting of a guanine to promote efficient labeling by T4 polynucleotide kinase [103]. The double stranded oligonucleotides were 5′-end labeled by T4 polynucleotide kinase (Thermo Scientific) and [γ-^32^P]ATP (222TBq/mmole), according to the manufacturer's instructions. The probe was purified on MicroSpin G-25 columns (GE Healthcare) and further processed as described in [104].

EMSAs were performed with 340 µg of purified HMG5His in 20 mM Tris (pH9.0), 500 mM NaCl, 5 mM β-mercaptoethanol, 5 mM MgCl_2_ and 5% glycerol supplemented with 1.25 µg of poly(dI-dC) in a total volume of 11 µl. Samples were incubated for 20 min on ice, labeled probe was added, and incubation was continued for 1 h on ice. For competition experiments, unlabeled double stranded oligonucleotides were added after incubating 20 min with poly(dI-dC) and incubated further for 15 min before adding the labeled probe.

Protein-DNA complexes were separated in polyacrylamide gel (6%) in 0.25× Tris-borate-EDTA buffer at 200 V per gel for ∼80 min. Radioactive probes were visualized using a Typhoon laser scanner (GE Healthcare).

## Supporting Information

Dataset S1 HMG-box domains used for Figure 2.
(TXT)

Dataset S2 Alignment of HMG-box domains used for Figure 2.
(TXT)

Figure S1 Comparative organization around *SpeSte11* orthologs in *P*. *anserina* and *N. crassa*. Orthologs were determined using FUNGIpath [52]. The gene names are enclosed in arrowed boxes indicating gene orientation. Orthologous genes are enclosed in boxes of identical color and connected with double arrows. Genes enclosed in white boxes do not have any ortholog in *P. anserina* or *N. crassa*. Gene sizes are not to scale. A: synteny in *P. anserina* and *N. crassa* for genes upstream and downstream of *PaHMG5* and *NCU09387*. The conserved synteny indicates that *PaHMG5* is the ortholog of *NCU09387*. B: organization of genes upstream and downstream of *NCU02326* in *N. crassa* and search for a synteny in *P. anserina*. The absence of a conserved synteny confirms that the ortholog of *NCU02326* is absent in *P. anserina*.
(TIFF)

Figure S2 Genomic Southern blots of HMG-box mutant strains probed with the hygromycin sequence. 1: DNA from *ΔPahmg6* transformants # 1 and # 2 digested with *Kpn*I. Both transformants displayed the expected pattern for homologous recombination. 2: DNA from *ΔPahmg2* transformants # 1 and # 2 digested with *Kpn*I. Only transformant # 2 displayed the expected pattern for homologous recombination. 3: DNA from *ΔPahmg3* transformants # 1 and # 2 digested with *Eco*RI. Both transformants displayed the expected pattern for homologous recombination. 4: DNA from *ΔPahmg5* transformants # 1 and # 2 digested with *Eco*RI. Both transformants displayed the expected pattern for homologous recombination. 5: DNA from *ΔPahmg6* transformants # 1 and # 2 digested with *Eco*RI. Both transformants displayed the expected pattern for homologous recombination. 6: DNA from *ΔPahmg4* transformants # 1 and # 2 digested with *Eco*RI. Only transformant #1 displayed the expected pattern for homologous recombination. 7: DNA from *Δkef1* transformant # 1 digested with *Eco*RI. This transformant displayed the expected pattern for homologous recombination. 8: DNA from *ΔPahmg7* transformants # 1 and # 2 digested with *Kpn*I. Both transformants displayed the expected pattern for homologous recombination.
(TIFF)

Figure S3 Electrophoretic mobility shift assays with PaHMG5 and mating-type target gene oligonucleotides. (A) Interaction of His tagged PaHMG5 (HMG5His) with probes corresponding to *mat−* mating-type target genes: *Pa_1_8290* (MFM), *Pa_1_9070* (PRE1) and *Pa_1_7350* (7350). The probe is indicated below each panel with the sequence of its core HMG-box binding site. The interaction of HMG5His with probe was analyzed without competitor and in the presence of competitor at 100 fold molar excess. (B) Interaction of His tagged PaHMG5 (HMG5His) with probes corresponding to *mat+* mating-type target genes: *Pa_2_2310* (MFP), *Pa_3_1710* (AOX), *Pa_4_3160* (PEPCK) and *Pa_1_24410* (24410). Legend as in (A).
(TIFF)

Figure S4 Electrophoretic mobility shift assays with PaHMG5 and HMG-box gene oligonucleotides. Interaction of His tagged PaHMG5 (HMG5His) with probes corresponding to HMG-box genes: *KEF1*, *mtHMG1*, *PaHMG2*, *PaHMG3*, *PaHMG4* and *PaHMG6*. Legend as in Figure S3 (A).
(TIFF)

Figure S5 Electrophoretic mobility shift reciprocal competition assays with PaHMG5 and HMG-box gene oligonucleotides. Legend as in Figure 8 (B). (A) Interaction of the HMG5His protein with the PaHMG2 probe and reciprocal competition between FMR1 and PaHMG2 oligonucleotides. HMG5His has a greater affinity for FMR1 than for PaHMG2 oligonucleotides, as indicated by the efficient exclusion of PaHMG2 probe by FMR1 competitor and the inefficient exclusion of FMR1 probe by PaHMG2 competitor. (B) Interaction of the HMG5His protein with the PaHMG3 probe and reciprocal competition between FMR1 and PaHMG3 oligonucleotides. HMG5His has a greater affinity for FMR1 than for PaHMG3 oligonucleotides, as indicated by the efficient exclusion of PaHMG3 probe by FMR1 competitor and the inefficient exclusion of FMR1 probe by PaHMG3 competitor. (C) Interaction of the HMG5His protein with the PaHMG4 probe and reciprocal competition between FMR1 and PaHMG4 oligonucleotides. HMG5His has a greater affinity for FMR1 than for PaHMG4 oligonucleotides, as indicated by the efficient exclusion of PaHMG4 probe by FMR1 competitor and the inefficient exclusion of FMR1 probe by PaHMG4 competitor. (D) Interaction of the HMG5His protein with the PaHMG6 probe and reciprocal competition between FMR1 and PaHMG6 oligonucleotides. HMG5His has a greater affinity for FMR1 than for PaHMG6 oligonucleotides, as indicated by the efficient exclusion of PaHMG6 probe by FMR1 competitor and the inefficient exclusion of FMR1 probe by PaHMG6 competitor.
(TIFF)

Table S1 Color scheme used for Jalview.
(DOC)

Table S2 Code and accession numbers for proteins shown in Figure 2.
(DOC)

Table S3 Relative quantification of HMG-box gene and mating-type target gene transcription in *ΔPahmg5* (*ΔPa_1_13940*) and *WT* strains.
(DOC)

Table S4 Relative quantification of HMG-box gene and mating-type target gene transcription in *ΔPahmg6* (*ΔPa_1_14230*) and *WT* strains.
(DOC)

Table S5 Relative quantification of HMG-box gene and mating-type target gene transcription in *ΔPahmg8* (*ΔPa_6_4110*) and *WT* strains.
(DOC)

Table S6 Relative quantification of HMG-box gene and mating-type target gene transcription in *Δkef1* (*ΔPahmg9*, *ΔPa_1_7190*) and *WT* strains.
(DOC)

Table S7 Relative quantification of HMG-box gene and mating-type target gene transcription in *Δmthmg1* (*ΔPa_1_13340*) and *WT* strains.
(DOC)

Table S8 Phenotype of *F. graminearum* strains deleted for HMGB genes (adapted from [43]).
(DOC)

Table S9 Oligonucleotide primers used for HMG-box gene deletion.
(DOC)

Table S10 Oligonucleotide primers used for HMG-box gene amplification.
(DOC)

Table S11 Complementation of *P. anserina* mutant strains.
(DOC)

Table S12 Oligonucleotide primers used for RT-qPCR.
(DOC)

Table S13 Normalization genes (REF) used for RT-qPCR.
(DOC)

Table S14 Oligonucleotides primers used for EMSA.
(DOC)
