# Supplementary figures and images for: A Network of HMG-box Transcription Factors Regulates Sexual Cycle in the Fungus Podospora anserina
Source: PLoS Genet. 2013 Jul 18;9(7):e1003642. doi: 10.1371/journal.pgen.1003642 (PMC3730723; doi:10.1371/journal.pgen.1003642)

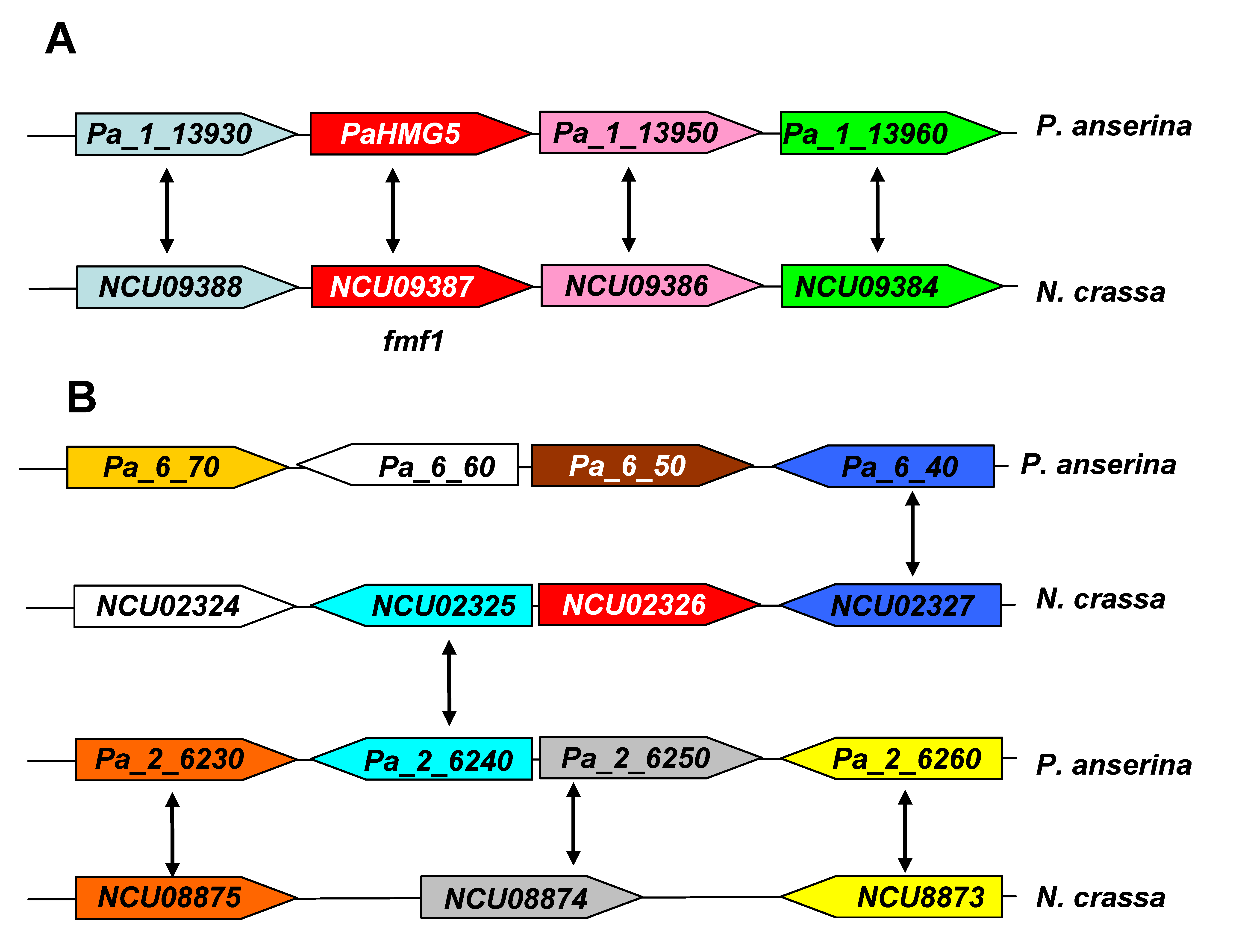

Supplement: Figure S1 — Comparative organization around SpeSte11 orthologs in P. anserina and N. crassa. Orthologs were determined using FUNGIpath [52]. The gene names are enclosed in arrowed boxes indicating gene orientation. Orthologous genes are enclosed in boxes of identical color and connected with double arrows. Genes enclosed in white boxes do not have any ortholog in P. anserina or N. crassa. Gene sizes are not to scale. A: synteny in P. anserina and N. crassa for genes upstream and downstream of PaHMG5 and NCU09387. The conserved synteny indicates that PaHMG5 is the ortholog of NCU09387. B: organization of genes upstream and downstream of NCU02326 in N. crassa and search for a synteny in P. anserina. The absence of a conserved synteny confirms that the ortholog of NCU02326 is absent in P. anserina. (TIFF) [file pgen.1003642.s003.tiff]

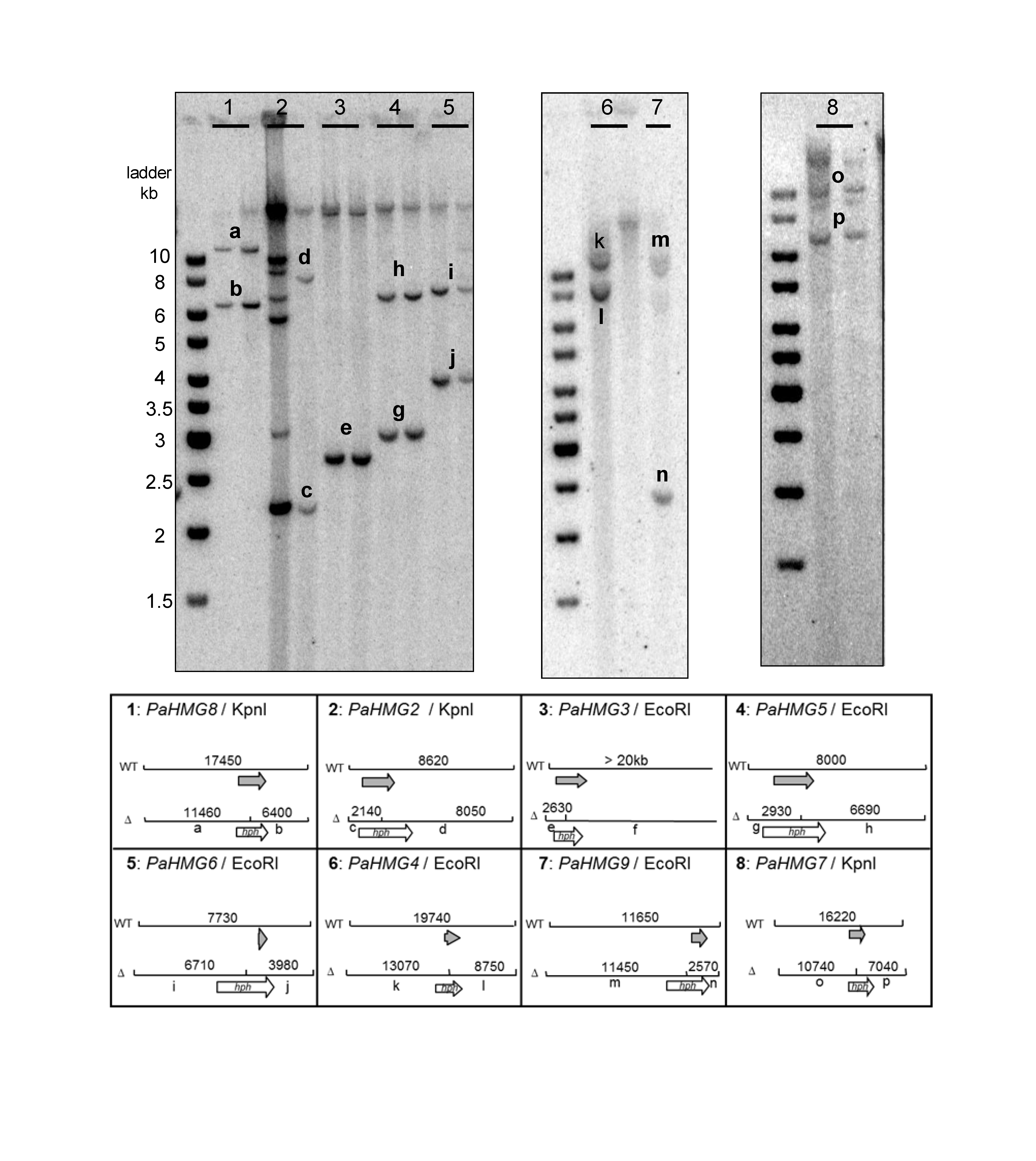

Supplement: Figure S2 — Genomic Southern blots of HMG-box mutant strains probed with the hygromycin sequence. 1: DNA from ΔPahmg6 transformants # 1 and # 2 digested with KpnI. Both transformants displayed the expected pattern for homologous recombination. 2: DNA from ΔPahmg2 transformants # 1 and # 2 digested with KpnI. Only transformant # 2 displayed the expected pattern for homologous recombination. 3: DNA from ΔPahmg3 transformants # 1 and # 2 digested with EcoRI. Both transformants displayed the expected pattern for homologous recombination. 4: DNA from ΔPahmg5 transformants # 1 and # 2 digested with EcoRI. Both transformants displayed the expected pattern for homologous recombination. 5: DNA from ΔPahmg6 transformants # 1 and # 2 digested with EcoRI. Both transformants displayed the expected pattern for homologous recombination. 6: DNA from ΔPahmg4 transformants # 1 and # 2 digested with EcoRI. Only transformant #1 displayed the expected pattern for homologous recombination. 7: DNA from Δkef1 transformant # 1 digested with EcoRI. This transformant displayed the expected pattern for homologous recombination. 8: DNA from ΔPahmg7 transformants # 1 and # 2 digested with KpnI. Both transformants displayed the expected pattern for homologous recombination. (TIFF) [file pgen.1003642.s004.tiff]

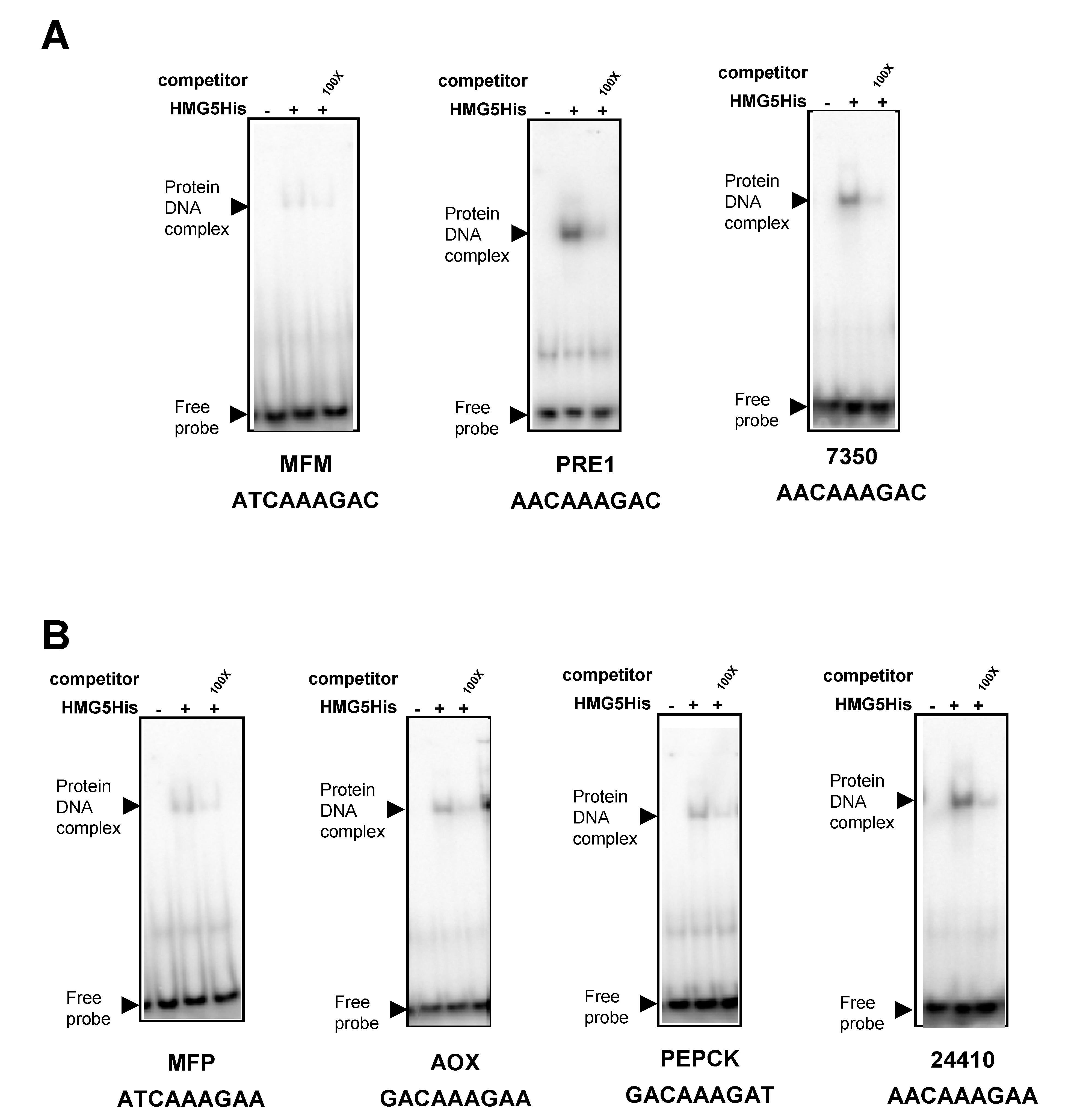

Supplement: Figure S3 — Electrophoretic mobility shift assays with PaHMG5 and mating-type target gene oligonucleotides. (A) Interaction of His tagged PaHMG5 (HMG5His) with probes corresponding to mat− mating-type target genes: Pa_1_8290 (MFM), Pa_1_9070 (PRE1) and Pa_1_7350 (7350). The probe is indicated below each panel with the sequence of its core HMG-box binding site. The interaction of HMG5His with probe was analyzed without competitor and in the presence of competitor at 100 fold molar excess. (B) Interaction of His tagged PaHMG5 (HMG5His) with probes corresponding to mat+ mating-type target genes: Pa_2_2310 (MFP), Pa_3_1710 (AOX), Pa_4_3160 (PEPCK) and Pa_1_24410 (24410). Legend as in (A). (TIFF) [file pgen.1003642.s005.tiff]

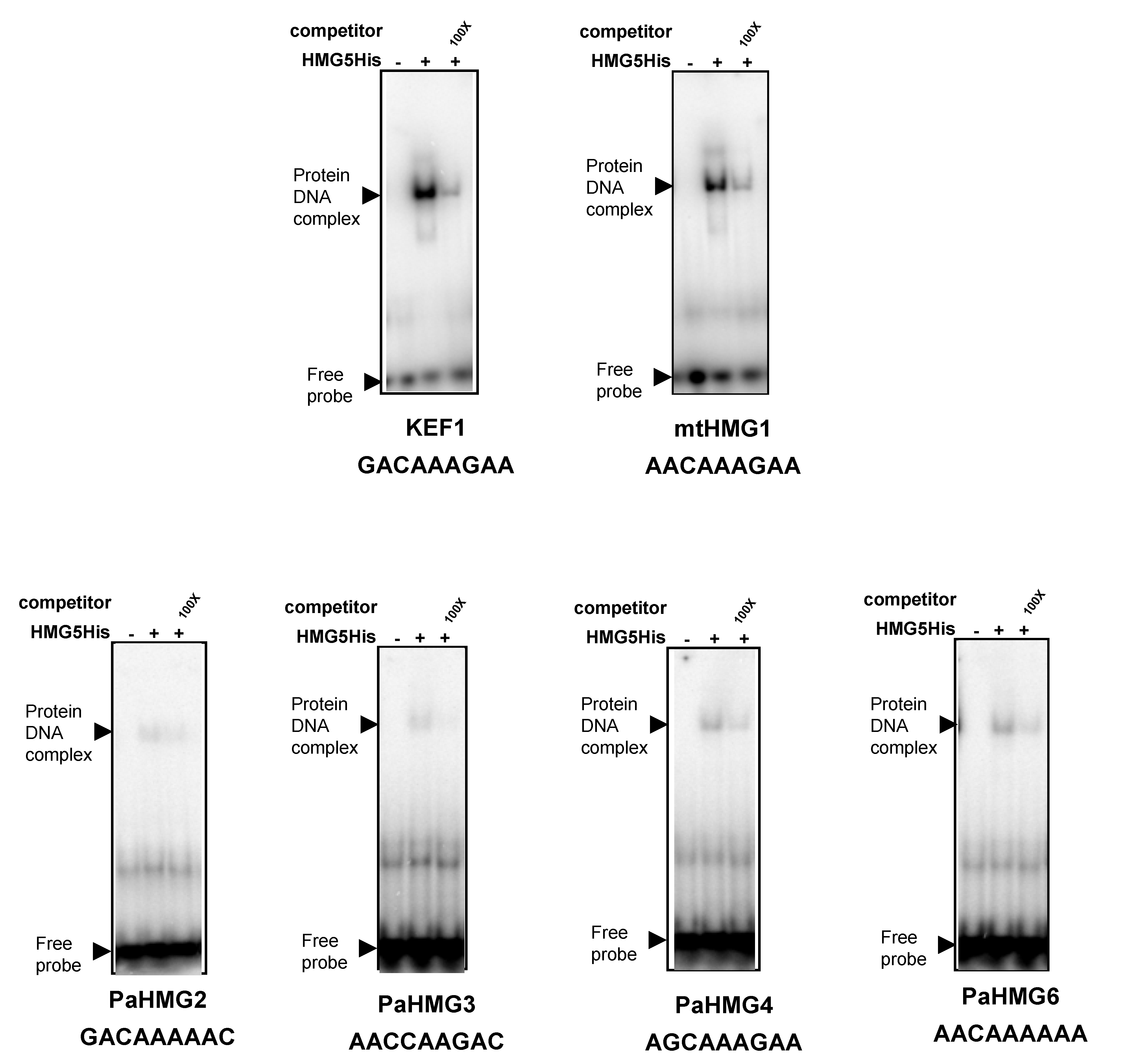

Supplement: Figure S4 — Electrophoretic mobility shift assays with PaHMG5 and HMG-box gene oligonucleotides. Interaction of His tagged PaHMG5 (HMG5His) with probes corresponding to HMG-box genes: KEF1, mtHMG1, PaHMG2, PaHMG3, PaHMG4 and PaHMG6. Legend as in Figure S3 (A). (TIFF) [file pgen.1003642.s006.tiff]

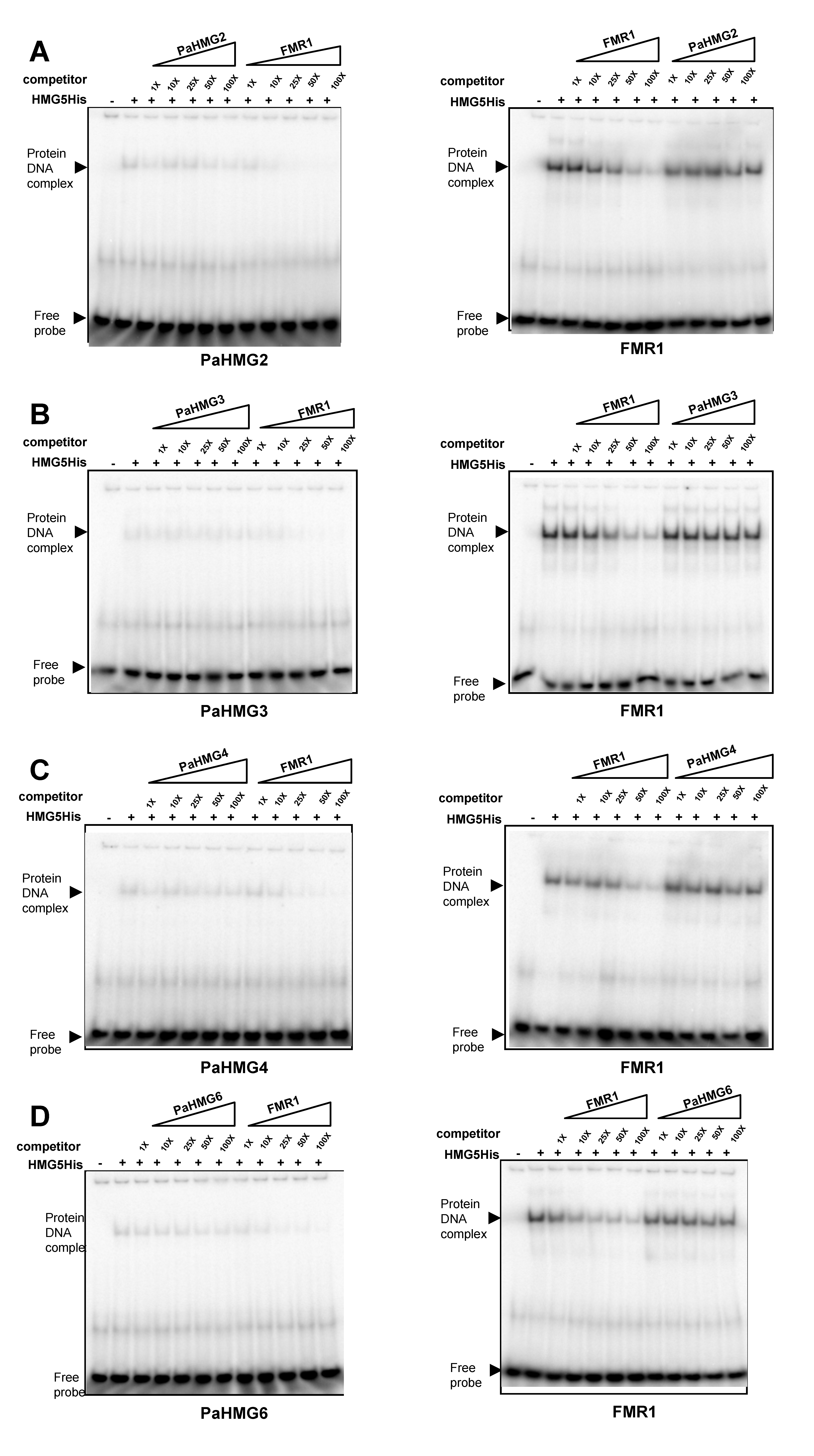

Supplement: Figure S5 — Electrophoretic mobility shift reciprocal competition assays with PaHMG5 and HMG-box gene oligonucleotides. Legend as in Figure 8 (B). (A) Interaction of the HMG5His protein with the PaHMG2 probe and reciprocal competition between FMR1 and PaHMG2 oligonucleotides. HMG5His has a greater affinity for FMR1 than for PaHMG2 oligonucleotides, as indicated by the efficient exclusion of PaHMG2 probe by FMR1 competitor and the inefficient exclusion of FMR1 probe by PaHMG2 competitor. (B) Interaction of the HMG5His protein with the PaHMG3 probe and reciprocal competition between FMR1 and PaHMG3 oligonucleotides. HMG5His has a greater affinity for FMR1 than for PaHMG3 oligonucleotides, as indicated by the efficient exclusion of PaHMG3 probe by FMR1 competitor and the inefficient exclusion of FMR1 probe by PaHMG3 competitor. (C) Interaction of the HMG5His protein with the PaHMG4 probe and reciprocal competition between FMR1 and PaHMG4 oligonucleotides. HMG5His has a greater affinity for FMR1 than for PaHMG4 oligonucleotides, as indicated by the efficient exclusion of PaHMG4 probe by FMR1 competitor and the inefficient exclusion of FMR1 probe by PaHMG4 competitor. (D) Interaction of the HMG5His protein with the PaHMG6 probe and reciprocal competition between FMR1 and PaHMG6 oligonucleotides. HMG5His has a greater affinity for FMR1 than for PaHMG6 oligonucleotides, as indicated by the efficient exclusion of PaHMG6 probe by FMR1 competitor and the inefficient exclusion of FMR1 probe by PaHMG6 competitor. (TIFF) [file pgen.1003642.s007.tiff]
