# Supplementary material for: A Network of HMG-box Transcription Factors Regulates Sexual Cycle in the Fungus Podospora anserina
Source: PLoS Genet. 2013 Jul 18;9(7):e1003642. doi: 10.1371/journal.pgen.1003642 (PMC3730723; doi:10.1371/journal.pgen.1003642)
Supplement: Table S1 — Color scheme used for Jalview. (DOC) [file pgen.1003642.s008.doc]

Table S1: colour scheme used for Jalview.

| Residue at position^a^ | Applied color | Rules for color (minimum %, residue group)^b^ |
| --- | --- | --- |
| A, I, L, M, F, W, V | BLUE | (+ 60%, WLVIMAFCHP) |
| R, K | RED | (+ 60%, KR), (+ 80%, K, R, Q) |
| N | GREEN | (+ 50%, N), (+ 85%, N, Y) |
| C | BLUE | (+ 60%, WLVIMAFCHP) |
| C | PINK | (100%, C) |
| Q | GREEN | (+ 60%, KR), (+ 50%, QE), (+ 85%, Q, E, K, R) |
| E | MAGENTA | (+ 60%, KR), (+ 50%, QE), (+ 85%, E, Q, D) |
| D | MAGENTA | (+ 60%, KR), (+ 85%, K, R, Q), (+ 50%, ED) |
| G | ORANGE | (+ 0%, G) |
| H, Y | CYAN | (+ 60%, WLVIMAFCHP), (+ 85% W, Y, A, C, P, Q, F, H, I, L, M, V) |
| P | YELLOW | (+ 0%, P) |
| S, T | GREEN | (+ 60%, WLVIMAFCHP), (+ 50%, TS), (+ 85%, S, T) |

^a^ if more than one residue is specified, the rules applied to each of these residues.

^b^ if a group of residues is concatenated together, such as 'WLVIMAFCHP', then any combination of these residues in total must meet or exceed the given percentage for the colour to be applied. For residues or residue groups separated by commas (*e*. *g*. W, Y, A, C, P, Q, F, H, I, L, M, V) at least one of these must by itself exceed the percentage.
