# Supplementary material for: A Network of HMG-box Transcription Factors Regulates Sexual Cycle in the Fungus Podospora anserina
Source: PLoS Genet. 2013 Jul 18;9(7):e1003642. doi: 10.1371/journal.pgen.1003642 (PMC3730723; doi:10.1371/journal.pgen.1003642)
Supplement: Table S2 — Code and accession numbers for proteins shown in Figure 2. (DOC) [file pgen.1003642.s009.doc]

**Table S2.** Code and accession numbers for proteins shown in Figure 2.

| **Group** | **Code** | **Protein name** | **Domain** | **Species** | **Accession number** |
| --- | --- | --- | --- | --- | --- |
| Sordariales | Podan1 | MAT1-1-1/FMR1 | α1 | *Podospora anserina* | CAA45519 |
|  | Podan2a | MAT1-2-1/FPR1 | MATA_HMG | *Podospora anserina* | CAA45520.1 |
|  | Podan3 | MAT1-1-3/SMR2 | MATA_HMG | *Podospora anserina* | CAA52051 |
|  | Podan2b | PaHMG8 | MATA_HMG | *Podospora anserina* | Pa_6_4110 |
|  | Podan2c | PaHMG5 | MATA_HMG | *Podospora anserina* | Pa_1_13940 |
|  | Podan5a | PaHMG6 | HMGB | *Podospora anserina* | Pa_1_14230 |
|  | Podan5b | PaHMG4 | HMGB | *Podospora anserina* | Pa_1_11050 |
|  | Podan5c | PaHMG2 | HMGB | *Podospora anserina* | Pa_1_7390 |
|  | Podan5d | mtHMG1 | HMGB | *Podospora anserina* | Pa_1_13340 |
|  | Podan5e | PaHMG3 | HMGB | *Podospora anserina* | Pa_1_9380 |
|  | Podan6b | PaHMG7 | HMG | *Podospora anserina* | Pa_5_8400 |
|  | Podan6a | PaHMG9/KEF1 | HMG | *Podospora anserina* | Pa_7_7190 |
|  | Neucr1 | MAT1-1-1/mat A-1 | α1 | *Neurospora crassa* | AAC37478 |
|  | Neucr2a | MAT1-2-1/mat a-1 | MATA_HMG | *Neurospora crassa* | AAA33598 |
|  | Neucr3 | MAT1-1-3/mat A-3 | MATA_HMG | *Neurospora crassa* | AAC37476 |
|  | Neucr2b |  | MATA_HMG | *Neurospora crassa* | NCU03481 |
|  | Neucr2c | FMF-1 | MATA_HMG | *Neurospora crassa* | NCU09387 |
|  | Neucr2d |  | MATA_HMG | *Neurospora crassa* | NCU02326 |
|  | Neucr5a |  | HMGB | *Neurospora crassa* | NCU09995 |
|  | Neucr5b |  | HMGB | *Neurospora crassa* | NCU03126 |
|  | Neucr5c |  | HMGB | *Neurospora crassa* | NCU02819 |
|  | Neucr5d |  | HMGB | *Neurospora crassa* | NCU02695 |
|  | Neucr5e |  | HMGB | *Neurospora crassa* | NCU09120 |
|  | Neucr6a |  | HMG | *Neurospora crassa* | NCU07568 |
|  | Neucr6b |  | HMG | *Neurospora crassa* | NCU06874 |
|  | Sorma1 | SMTA-1 | α1 | *Sordaria macrospora* | CAA71623 |
|  | Sorma2 | SMTa-1 | MATA_HMG | *Sordaria macrospora* | CAA71624.1 |
| Magnaporthales | Magor1 | MAT1-1-1 | α1 | *Magnaporthe oryzae* | BAC65083.1 |
|  | Magor2 | MAT1-2-1 | MATA_HMG | *Magnaporthe oryzae* | BAC65094.1 |
| Diaporthales | CryPa1 | MAT1-1-1 | α1 | *Cryphonectria parasitica* | AAK83346 |
|  | CryPa3 | MAT1-1-3 | MATA_HMG | *Cryphonectria parasitica* | AAK83344.1 |
| Hypocreales | Fusac3 | MAT1-1-3 | MATA_HMG | *Fusarium acaciae-mearnsii* | ABE98373.1 |
|  | Gibfu1 | MAT1-1-1 | α1 | *Gibberella fujikuroi* | AAC71055 |
|  | Gibfu3 | MAT1-1-3 | MATA_HMG | *Gibberella fujikuroi* | AAC71053.1 |
|  | Gibze1 | MAT1-1-1 | α1 | *Gibberella zeae* | AAG42809 |
|  | Gibze3 | MAT1-1-3 | MATA_HMG | *Gibberella zeae* | AAG42812 |
| incertae sedis | Verda2 | MAT1-2-1 | MATA_HMG | *Verticillium dahliae* | BAG12301.1 |
| Helotiales | Botfu5 | HMG | HMGB | *Botryotinia fuckeliana* | XP_001548220 |
|  | Pyrbr1 | MAT1-1-1/Pad1 | α1 | *Pyrenopeziza brassicae* | CAA06844 |
|  | Pyrbr3 | MAT1-1-3/phb1 | MATA_HMG | *Pyrenopeziza brassicae* | CAA06846.1 |
|  | Rhyse1 | MAT1-1-1 | α1 | *Rhynchosporium secalis* | CAD71141.1 |
| Eurotiales | Aspfu1 | MAT1-1-1 | α1 | *Aspergillus fumigatus* | AAX83123.1 |
|  | Aspfu2 |  | MATA_HMG | *Aspergillus fumigatus* | XP_751745.1 |
|  | Aspni1 | MAT1-1/MATB | α1 | *Aspergillus nidulans* | EAA63189.1 |
|  | Aspni2a | MAT1-2/MATA | MATA_HMG | *Aspergillus nidulans* | AN4734 |
|  | Aspni2b |  | MATA_HMG | *Aspergillus nidulans* | AN1962 |
|  | Aspni2c |  | MATA_HMG | *Aspergillus nidulans* | AN3667 |
|  | Aspni2d |  | MATA_HMG | *Aspergillus nidulans* | AN3549 |
|  | Aspni5a |  | HMGB | *Aspergillus nidulans* | AN2885 |
|  | Aspni5b |  | HMGB | *Aspergillus nidulans* | AN10103 |
|  | Aspni5c |  | HMGB | *Aspergillus nidulans* | AN1267 |
|  | Aspni6 |  | HMG | *Aspergillus nidulans* | AN3580 |
|  | Penma1 | MAT1-1-1/MAT-1 | α1 | *Penicillium marneffei* | ABC68484.1 |
|  | Penma2 |  | MATA_HMG | *Penicillium marneffei* | XP_002151220.1 |
| Onygenales | Ajeca1 | MAT1-1-1 | α1 | *Ajellomyces capsulatus* | ABO87596.1 |
|  | Ajeca2 | MAT1-2-1 | MATA_HMG | *Ajellomyces capsulatus* | EER39720.1 |
| Pleosporales | Altal1 | MAT1-1-1 | α1 | *Alternaria alternata* | BAA75907.1 |
|  | Altal2 | MAT1-2-1 | MATA_HMG | *Alternaria alternata* | BAA75903.1 |
|  | Altbr1 | MAT1-1-1 | α1 | *Alternaria brassicicola* | AAK85542.1 |
|  | Bipsa2 | MAT1-2-1 | MATA_HMG | *Bipolaris sacchari* | CAA65081.1 |
|  | Coche1 | MAT1-1-1 | α1 | *Cochliobolus heterostrophus* | CAA48465 |
|  | Coche2a | MAT1-2-1 | MATA_HMG | *Cochliobolus heterostrophus* | CAA48464.1 |
|  | Cocho2 | MAT1-2/1 | MATA_HMG | *Cochliobolus homomorphus* | AAD33441.1 |
|  | Coche2b |  | MATA_HMG | *Cochliobolus heterostrophus* | 29997 |
|  | Coche2c |  | MATA_HMG | *Cochliobolus heterostrophus* | 29252 |
|  | Coche5e |  | HMGB | *Cochliobolus heterostrophus* | 105755 |
|  | Coche5c |  | HMGB | *Cochliobolus heterostrophus* | 14121 |
|  | Coche5d |  | HMGB | *Cochliobolus heterostrophus* | 31679 |
|  | Coche5b |  | HMGB | *Cochliobolus heterostrophus* | 33388 |
|  | Coche5a |  | HMGB | *Cochliobolus heterostrophus* | 62751 |
|  | Coche6 |  | HMG | *Cochliobolus heterostrophus* | 27893 |
|  | Pyrte2 | MAT1-2-1 | MATA_HMG | *Pyrenophora teres* | AAY35017 |
|  | Stesa1 | MAT1-1 | α1 | *Stemphylium sarciniforme* | AAR04460 |
| Dothideales | Dotpi2 | MAT1-2-1 | MATA_HMG | *Dothistroma pini* | ABK91353 |
|  | Mycgr1 | MAT1-1-1 | α1 | *Mycosphaerella graminicola* | AAL30838 |
|  | Mycgr2 | MAT1-2-1 | MATA_HMG | *Mycosphaerella graminicola* | AAL30836.1 |
| Saccharomycotina | Canal1 | MTL α1 | α1 | *Candida albicans* | XP_714749 |
|  | Canal2 | Rfg1p | MATA_HMG | *Candida albicans* | XP_715804.1 |
|  | Lacth1 | MATα1 | α1 | *Lachancea thermotolerans* | XP_002554225.1 |
|  | Sacce1 | Matα1p | α1 | *Saccharomyces cerevisiae* | EDN62161.1 |
|  | Sacce2 | Rox1p | MATA_HMG | *Saccharomyces cerevisiae* | NP_015390.1 |
|  | Sacce5a | NHP6A | HMGB | *Saccharomyces cerevisiae* | EDN61184 |
|  | Sacce5b | NHP6B | HMGB | *Saccharomyces cerevisiae* | NP_009647 |
|  | Sacce5c | Nhp10p/Hmo2p | HMGB | *Saccharomyces cerevisiae* | [NP_010282.1](http://www.ncbi.nlm.nih.gov/protein/6320202?report=genbank&log$=prottop&blast_rank=1&RID=MJRHBMMF01S) |
|  | Sacce5d | Hmo1p | HMGB | *Saccharomyces cerevisiae* | [NP_010459.1](http://www.ncbi.nlm.nih.gov/protein/6320379?report=genbank&log$=prottop&blast_rank=1&RID=MJRNNUNB01N) |
|  | Sacce5e  Sacce5f | Ixr1p | HMGB  HMGB | *Saccharomyces cerevisiae* | [NP_012893.1](http://www.ncbi.nlm.nih.gov/protein/6322820?report=genbank&log$=prottop&blast_rank=1&RID=MJRRVWM001N) |
|  | Sacce5g  Sacce5h | Abf2p | HMGB  HMGB | *Saccharomyces cerevisiae* | [NP_013788.1](http://www.ncbi.nlm.nih.gov/protein/6323717?report=genbank&log$=prottop&blast_rank=1&RID=MJRVNABJ01S) |
|  | Zygro1 | MAT α1 | α1 | *Zygosaccharomyces rouxii* | XP_002497889.1 |
| Taphrinomycotina | Pneca2 | STE11 | MATA_HMG | *Pneumocystis carinii* | Q870J1 |
|  | Schja2 | STE11 | MATA_HMG | *Schizosaccharomyces japonicus* | XP_002175130 |
|  | Schpo2c | STE11 | MATA_HMG | *Schizosaccharomyces pombe* | CAA77507.1 |
|  | Schpo6 | Pc | HMG | *Schizosaccharomyces pombe* | P10841 |
|  | Schpo2a | mat-Mc | MATA_HMG | [*Schizosaccharomyces pombe*](http://fungalgenomes.org/w/index.php?title=Schizosaccharomyces_pombe&action=edit&redlink=1) | [NP_595867.1](http://www.ncbi.nlm.nih.gov/protein/19112659?report=genbank&log$=prottop&blast_rank=1&RID=MJTDRCX101S) |
|  | Schpo5a |  | HMGB | [*Schizosaccharomyces pombe*](http://fungalgenomes.org/w/index.php?title=Schizosaccharomyces_pombe&action=edit&redlink=1) | [NP_595672.1](http://www.ncbi.nlm.nih.gov/protein/19112464?report=genbank&log$=prottop&blast_rank=1&RID=MJS8RGYG01S) |
|  | Schpo5d | Nht1 | HMGB | [*Schizosaccharomyces pombe*](http://fungalgenomes.org/w/index.php?title=Schizosaccharomyces_pombe&action=edit&redlink=1) | [NP_593259.1](http://www.ncbi.nlm.nih.gov/protein/19114171?report=genbank&log$=prottop&blast_rank=1&RID=MJSAY1FG01N) |
|  | Schpo5c | Nhp6 | HMGB | [*Schizosaccharomyces pombe*](http://fungalgenomes.org/w/index.php?title=Schizosaccharomyces_pombe&action=edit&redlink=1) | [NP_593314.1](http://www.ncbi.nlm.nih.gov/protein/19114226?report=genbank&log$=prottop&blast_rank=1&RID=MJSFPWJN01S) |
|  | Schpo5b | Cmb1 | HMGB | [*Schizosaccharomyces pombe*](http://fungalgenomes.org/w/index.php?title=Schizosaccharomyces_pombe&action=edit&redlink=1) | [NP_593693.1](http://www.ncbi.nlm.nih.gov/protein/19114605?report=genbank&log$=prottop&blast_rank=1&RID=MJSJXH4501S) |
|  | Schpo7a | Lsd1 | HMG | [*Schizosaccharomyces pombe*](http://fungalgenomes.org/w/index.php?title=Schizosaccharomyces_pombe&action=edit&redlink=1) | [NP_595398.1](http://www.ncbi.nlm.nih.gov/protein/19112190?report=genbank&log$=prottop&blast_rank=1&RID=MJSSERCT01S) |
|  | Schpo7b |  | HMG | [*Schizosaccharomyces pombe*](http://fungalgenomes.org/w/index.php?title=Schizosaccharomyces_pombe&action=edit&redlink=1) | [NP_595970.1](http://www.ncbi.nlm.nih.gov/protein/19112762?report=genbank&log$=prottop&blast_rank=1&RID=MJS4J51901S) |
| Basidiomycota | Ustma2b | Prf1 | MATA_HMG | *Ustilago maydis* | AAC32736 |
|  | Ustma2c | Hmg3 / Rop1 | MATA_HMG | *Ustilago maydis* | UM01457 |
|  | Ustma2d |  | MATA_HMG | *Ustilago maydis* | UM06025 |
|  | Ustma5d  Ustma5e |  | HMGB  HMGB | *Ustilago maydis* | UM01390 |
|  | Ustma6  Ustma5f |  | HMG  HMGB | *Ustilago maydis* | UM04007 |
|  | Ustma5c |  | HMGB | *Ustilago maydis* | UM06160 |
|  | Ustma5a |  | HMGB | *Ustilago maydis* | UM02364 |
|  | Ustma5b |  | HMGB | *Ustilago maydis* | UM00808 |
|  | Copci2b | pcc1 | MATA_HMG | *Coprinopsis cinerea* | CC1G_07392 |
|  | Copci5b |  | HMGB | *Coprinopsis cinerea* | CC1G_01334 |
|  | Copci5a |  | HMGB | *Coprinopsis cinerea* | CC1G_11882 |
|  | Copci2c |  | HMG | *Coprinopsis cinerea* | CC1G_04121 |
|  | Copci2d |  | HMG | *Coprinopsis cinerea* | CC1G_07470 |
|  | Copci2e |  | HMG | *Coprinopsis cinerea* | CC1G_11980 |
|  | Copci2f |  | HMG | *Coprinopsis cinerea* | CC1G_07876 |
|  | Copci2g |  | HMG | *Coprinopsis cinerea* | CC1G_07859 |
|  | Copci2h |  | HMG | *Coprinopsis cinerea* | CC1G_03744 |
|  | Copci2i |  | HMG | *Coprinopsis cinerea* | CC1G_08141 |
|  | Copci2j |  | HMG | *Coprinopsis cinerea* | CC1G_13994 |
|  | Copci2k |  | HMG | *Coprinopsis cinerea* | CC1G_00255 |
|  | Copci2l |  | HMG | *Coprinopsis cinerea* | CC1G_06994 |
| Zygomycota | Phybl8 | SexM | HMG | *Phycomyces blakesleeanus* | ABX27909.1 |
|  | Phybl9 | SexP | HMG | *Phycomyces blakesleeanus* | ABX27912.1 |
| Microsporidia | Antlo7 | HMG | HMG | *Antonospora locustae* | ACI87876.1 |
|  | Entbi7 | Sex locus | HMG | *Enterocytozoon bieneusi* | ACI87872 |
|  | Enccu7 | Sex locus | HMG | *Encephalitozoon cuniculi* | NP_585883 |
| Animalia | Ailme4 | hypothetical | SOX | *Ailuropoda melanoleuca* | EFB23328 |
|  | Anoga4 | AGAP003896-PA | SOX | *Anopheles gambiae* | XP_001230616.1 |
|  | Caeel5 | HMG-4 | HMGB | *Caenorhabditis elegans* | NP_498633 |
|  | Cerel4 | SRY | SOX | *Cervus elaphus yarkandensis* | ABK91721 |
|  | Ciosa4 | TF | SOX | *Ciona savignyi* | NP_001071831.1 |
|  | Culqu4 | Pangolin | SOX | *Culex quinquefasciatus* | XP_001864781 |
|  | Danre4 | TF-7 | SOX | *Danio rerio* | AAI63927 |
|  | Danre5a | RNA Polymerase 1 | HMGB | *Danio rerio* | CAQ14015 |
|  | Danre5b | TF | HMGB | *Danio rerio* | NP_957297 |
|  | Danre5c | TF | HMGB | *Danio rerio* | NP_957297.1 |
|  | Drome4a | Pangolin | SOX | *Drosophila melanogaster* | NP_001014685.1 |
|  | Drome4b | bobby sox | SOX | *Drosophila melanogaster* | NP_001027087.1 |
|  | Homsa5a | RNA Polymerase 1 | HMGB | *Homo sapiens* | EAW51616 |
|  | Homsa5b | TF1 | HMGB | *Homo sapiens* | NP_055048.1 |
|  | Homsa5c | HMG | HMGB | *Homo sapiens* | 2CS1_A |
|  | Musmu4a | lymphoid enhancer | SOX | *Mus musculus* | EDL12207.1 |
|  | Musmu4b | SOX-1 | SOX | *Mus musculus* | NP_033259.2 |
|  | Musmu4c | HMG | SOX | *Mus musculus* | NP_694878.2 |
|  | Strpu4 | Tcf/Lef | SOX | *Strongylocentrotus purpuratus* | NP_999640.1 |
|  | Takru4 | SOX8b | SOX | *Takifugu rubripes* | AAQ18506 |
|  | Xenla4 | XTCF-3b | SOX | *Xenopus laevis* | CAA67689 |
|  | Xenla5a | TF1-B | HMGB | *Xenopus laevis* | NP_001079429 |
|  | Xenla5b | ubtf-b | HMGB | *Xenopus laevis* | AAH42232 |
|  | Xenla5c | ubtf-b | HMGB | *Xenopus laevis* | AAH42232.1 |
| Planta | Arath5 | HMG | HMGB | *Arabidopsis thaliana* | AAK43965.1 |
