# Supplementary material for: A Network of HMG-box Transcription Factors Regulates Sexual Cycle in the Fungus Podospora anserina
Source: PLoS Genet. 2013 Jul 18;9(7):e1003642. doi: 10.1371/journal.pgen.1003642 (PMC3730723; doi:10.1371/journal.pgen.1003642)
Supplement: Table S3 — Relative quantification of HMG-box gene and mating-type target gene transcription in ΔPahmg5 (ΔPa_1_13940) and WT strains. (DOC) [file pgen.1003642.s010.doc]

**Table S3.** Relative quantification of HMG-box gene and mating-type target gene transcription in *ΔPahmg5* (*ΔPa_1_13940*) and *WT* strains.

| Mating-type | Gene number | Gene name or function | fold change in mutant^a^ | Std. Error | 95% C.I. | p-value | Result^b^ |
| --- | --- | --- | --- | --- | --- | --- | --- |
| *mat+* | Pa_1_13340 | *mtHMG1* | 0.97 | 0.91 - 1.0 | 0.85 – 1.0 | 0.26 | N/S |
|  | Pa_1_14230 | *PaHMG6* | 1.0 | 0.97 – 1.1 | 0.93 – 1.1 | 0.25 | N/S |
|  | Pa_6_4110 | *PaHMG8* | 0.3 | 0.24 – 0.38 | 0.23 – 0.43 | 0.001 | down |
|  | Pa_7_7190 | *PaHMG9/KEF1* | 1.1 | 0.70 – 1.8 | 0.6 – 2.9 | 0.56 | N/S |
|  | Pa_1_20590 | *FPR1* | 0.25 | 0.20 – 0.30 | 0.18 – 0.37 | 0.006 | down |
|  | Pa_2_2310 | *MFP* | 0.0004 | 0.000 – 0.001 | 0.000 – 0.001 | 0.004 | down |
|  | Pa_4_1380 | *PRE2* | 0.26 | 0.24 – 0.30 | 0.21 -0.32 | 0.002 | down |
|  | Pa_4_3858 | Unknown function | 0.03 | 0.02 – 0.04 | 0.02 – 0.05 | 0.002 | down |
|  | Pa_1_24410 | SAM  methyl transferase | 0.0002 | 0.000- 0.000 | 0.000- 0.000 | 0.002 | down |
|  | Pa_5_9770 | *PAG* | 0.34 | 0.21 – 0.51 | 0.18 – 0.61 | 0.008 | down |
|  | Pa_3_1710 | *AOX* | 0.5 | 0.45 – 0.56 | 0.43 – 0.60 | 0.007 | down |
|  | Pa_4_3160 | *PEPCK* | 1.1 | 0.90 – 1.4 | 0.82 – 1.6 | 0.33 | N/S |
|  | Pa_4_80 | Methyl-transferase | 1.1 | 0.95 – 1.3 | 0.83 – 1.6 | 0.22 | N/S |
| mat- | Pa_1_13340 | *mtHMG1* | 1.1 | 0.95 - 1.3 | 0.88 - 1.4 | 0.14 | N/S |
|  | Pa_1_14230 | *PaHMG6* | 1.1 | 0.86 – 1.3 | 0.8 – 1.5 | 0.38 | N/S |
|  | Pa_6_4110 | *PaHMG8* | 0.36 | 0.3 – 0.42 | 0.26 – 0.45 | 0 | down |
|  | Pa_7_7190 | *PaHMG9/KEF1* | 0.76 | 0.52 – 1.1 | 0.38 – 1.5 | 0.14 | N/S |
|  | N/A | *FMR1* | 0.1 | 0.08 – 0.11 | 0.08 – 0.13 | 0.000 | down |
|  | Pa_1_8290 | *MFM* | 0.004 | 0.003 – 0.005 | 0.003 – 0.007 | 0.001 | down |
|  | Pa_7_9070 | *PRE1* | 0.13 | 0.10 – 0.18 | 0.08 – 0.22 | 0.001 | down |
|  | Pa_6_7350 | protease | 0.005 | 0.004 – 0.007 | 0.003 – 0.008 | 0.001 | down |

^a^: the fold-change is the ratio of cDNA in *ΔPahmg5* strain to *WT* (Materials and Methods).

^b^: transcription in *ΔPahmg5* strains*.* N/S: not significant.
