# Supplementary material for: A Network of HMG-box Transcription Factors Regulates Sexual Cycle in the Fungus Podospora anserina
Source: PLoS Genet. 2013 Jul 18;9(7):e1003642. doi: 10.1371/journal.pgen.1003642 (PMC3730723; doi:10.1371/journal.pgen.1003642)
Supplement: Table S4 — Relative quantification of HMG-box gene and mating-type target gene transcription in ΔPahmg6 (ΔPa_1_14230) and WT strains. (DOC) [file pgen.1003642.s011.doc]

**Table S4.** Relative quantification of HMG-box gene and mating-type target gene transcription in *ΔPahmg6* (*ΔPa_1_14230*) and *WT* strains.

| Mating-type | Gene number | Gene name or function | qPCR fold change^a^ | Std. Error | 95% C.I. | p-value | Result^b^ |
| --- | --- | --- | --- | --- | --- | --- | --- |
| *mat+* | Pa_1_13340 | *mtHMG1* | 0.72 | 0.66 – 0.76 | 0.61 – 0.81 | 0.001 | down |
|  | Pa_1_13940 | *PaHMG5* | 0.09 | 0.08 – 0.11 | 0.07 – 0.14 | 0 | down |
|  | Pa_6_4110 | *PaHMG8* | 0.12 | 0.1 – 0.17 | 0.08 – 0.22 | 0 | down |
|  | Pa_7_7190 | *PaHMG9/KEF1* | 1.1 | 0.65 – 2.6 | 0.5 – 4.3 | 0.8 | N/S |
|  | Pa_1_20590 | *FPR1* | 0.73 | 0.6- 0.95 | 0.53 – 1.1 | 0.026 | N/S |
|  | Pa_2_2310 | *MFP* | 0.07 | 0.05 – 0.10 | 0.05 – 0.15 | 0 | down |
|  | Pa_4_1380 | *PRE2* | 0.14 | 0.12 – 0.16 | 0.11 – 0.17 | 0 | down |
|  | Pa_4_3858 | Unknown function | 0.38 | 0.27 – 0.54 | 0.20 – 0.64 | 0 | down |
|  | Pa_1_24410 | SAM  methyl transferase | 0.12 | 0.09 – 0.16 | 0.076 – 0.22 | 0 | down |
|  | Pa_5_9770 | *PAG* | 0.075 | 0.05 – 0.10 | 0.04 – 0.11 | 0 | down |
|  | Pa_3_1710 | *AOX* | 3.9 | 3.2 – 5.0 | 2.8 – 5.8 | 0.008 | up |
|  | Pa_4_3160 | *PEPCK* | 0.46 | 0.33 – 0.57 | 0.28 – 0.62 | 0.003 | down |
|  | Pa_4_80 | Methyl-transferase | 16 | 12 - 22 | 11 - 28 | 0.007 | up |
| mat- | Pa_1_13340 | *mtHMG1* | 0.73 | 0.67 – 0.81 | 0.65 – 0.87 | 0.002 | down |
|  | Pa_1_13940 | *PaHMG5* | 0.07 | 0.04 – 0.11 | 0.03 – 0.13 | 0.004 | down |
|  | Pa_6_4110 | *PaHMG8* | 0.19 | 0.14 – 0.27 | 0.10 – 0.38 | 0.008 | down |
|  | Pa_7_7190 | *PaHMG9/KEF1* | 1.17 | 0.91 – 1.57 | 0.78 – 1.8 | 0.22 | N/S |
|  | N/A | *FMR1* | 0.11 | 0.08 – 0.18 | 0.07 – 0.21 | 0.005 | down |
|  | Pa_1_8290 | *MFM* | 0.06 | 0.04 – 0.10 | 0.03 – 0.14 | 0.005 | down |
|  | Pa_7_9070 | *PRE1* | 0.12 | 0.06 – 0.27 | 0.05 – 0.38 | 0.002 | down |
|  | Pa_6_7350 | protease | 0.14 | 0.07 – 0.20 | 0.05 – 0.25 | 0.003 | down |

^a^: the fold-change is the ratio of cDNA in *ΔPahmg6* strain to *WT* (Materials and Methods).

^b^: transcription in *ΔPahmg6* strains*.* N/S: not significant.
