# Supplementary material for: A Network of HMG-box Transcription Factors Regulates Sexual Cycle in the Fungus Podospora anserina
Source: PLoS Genet. 2013 Jul 18;9(7):e1003642. doi: 10.1371/journal.pgen.1003642 (PMC3730723; doi:10.1371/journal.pgen.1003642)
Supplement: Table S5 — Relative quantification of HMG-box gene and mating-type target gene transcription in ΔPahmg8 (ΔPa_6_4110) and WT strains. (DOC) [file pgen.1003642.s012.doc]

**Table S5.** Relative quantification of HMG-box gene and mating-type target gene transcription in *ΔPahmg8* (*ΔPa_6_4110*) and *WT* strains.

| Mating-type | Gene number | Gene name or function | fold change in mutant^a^ | Std. Error | 95% C.I. | p-value | Result^b^ |
| --- | --- | --- | --- | --- | --- | --- | --- |
| *mat+* | Pa_1_13340 | *mtHMG1* | 1.1 | 1 – 1.2 | 0.94 – 1.3 | 0.07 | N/S |
|  | Pa_1_13940 | *PaHMG5* | 1.3 | 0.98 – 1.5 | 0.84 – 1.6 | 0.035 | N/S |
|  | Pa_1_14230 | *PaHMG6* | 1.0 | 0.95 – 1.0 | 0.89 – 1.1 | 0.63 | N/S |
|  | Pa_7_7190 | *PaHMG9/KEF1* | 0.9 | 0.5 – 1.4 | 0.3 – 1.8 | 0.756 | N/S |
|  | Pa_1_20590 | *FPR1* | 0.45 | 0.32 – 0.58 | 0.28 – 0.77 | 0.003 | down |
|  | Pa_2_2310 | *MFP* | 0.25 | 0.20 – 0.36 | 0.19 – 0.43 | 0.007 | down |
|  | Pa_4_1380 | *PRE2* | 0.24 | 0.21 – 0.27 | 0.18 – 0.3 | 0 | down |
|  | Pa_4_3858 | Unknown function | 0.35 | 0.24 – 0.51 | 0.19 – 0.70 | 0.007 | down |
|  | Pa_1_24410 | SAM  methyl transferase | 0.72 | 0.6 – 0.9 | 0.54 – 1.0 | 0.019 | N/S |
|  | Pa_5_9770 | *PAG* | 2 | 1.28 – 2.8 | 0.85 – 3.2 | 0.019 | up |
|  | Pa_3_1710 | *AOX* | 0.79 | 0.60 – 1.0 | 0.57 – 1.18 | 0.06 | N/S |
|  | Pa_4_3160 | *PEPCK* | 1.3 | 0.77 – 1.7 | 0.7 – 1.7 | 0.15 | N/S |
|  | Pa_4_80 | Methyl-transferase | 1 | 0.45 – 2.2 | 0.38 – 3.6 | 0.98 | N/S |
| mat- | Pa_1_13340 | *mtHMG1* | 1.0 | 0.96 – 1.13 | 0.93 – 1.25 | 0.48 | N/S |
|  | Pa_1_13940 | *PaHMG5* | 1.34 | 1.15 – 1.5 | 0.98 – 1.7 | 0.016 | N/S |
|  | Pa_1_14230 | *PaHMG6* | 1.1 | 1.0 – 1.3 | 094 – 1.38 | 0.025 | N/S |
|  | Pa_7_7190 | *PaHMG9/KEF1* | 1.4 | 1.17 – 1.83 | 0.89 – 2.18 | 0.025 | N/S |
|  | N/A | *FMR1* | 0.45 | 0.38 – 0.59 | 0.35 – 0.62 | 0.007 | down |
|  | Pa_1_8290 | *MFM* | 0.60 | 0.51 – 0.70 | 0.45 – 0.77 | 0.008 | down |
|  | Pa_7_9070 | *PRE1* | 0.1 | 0.06 – 0.16 | 0.04 – 0.21 | 0.006 | down |
|  | Pa_6_7350 | protease | 1.5 | 1.4 – 1.7 | 1.2 – 1.9 | 0.007 | down |

^a^: the fold-change is the ratio of cDNA in *ΔPahmg8* strain to *WT* (Materials and Methods).

^b^: transcription in *ΔPahmg8* strains*.* N/S: not significant.
