# Supplementary material for: A Network of HMG-box Transcription Factors Regulates Sexual Cycle in the Fungus Podospora anserina
Source: PLoS Genet. 2013 Jul 18;9(7):e1003642. doi: 10.1371/journal.pgen.1003642 (PMC3730723; doi:10.1371/journal.pgen.1003642)
Supplement: Table S7 — Relative quantification of HMG-box gene and mating-type target gene transcription in Δmthmg1 (ΔPa_1_13340) and WT strains. (DOC) [file pgen.1003642.s014.doc]

**Table S7.** Relative quantification of HMG-box gene and mating-type target gene transcription in *Δmthmg1* (*ΔPa_1_13340*) and *WT* strains.

| Mating-type | Gene number | Gene name or function | fold change in mutant^a^ | Std. Error | 95% C.I. | p-value | Result^b^ |
| --- | --- | --- | --- | --- | --- | --- | --- |
| *mat+* | Pa_1_13940 | *PaHMG5* | 1.5 | 1.2 – 1.8 | 1.1 – 1.9 | 0.004 | up |
|  | Pa_1_14230 | *PaHMG6* | 1.2 | 1.1 – 1.4 | 1.0 – 1.5 | 0.007 | N/S |
|  | Pa_6_4110 | *PaHMG8* | 0.8 | 0.6 – 0.9 | 0.5 – 1.0 | 0.03 | N/S |
|  | Pa_7_7190 | *PaHMG9/KEF1* | 0.5 | 0.4 – 0.7 | 0.3 – 0.8 | 0.005 | down |
|  | Pa_1_20590 | *FPR1* | 1.0 | 0.9 – 1.2 | 0.8 – 1.2 | 0.5 | N/S |
|  | Pa_2_2310 | *MFP* | 0.32 | 0.2 – 0.4 | 0.15 – 0.5 | 0.004 | down |
|  | Pa_4_1380 | *PRE2* | 0.30 | 0.2 – 0.4 | 0.2 – 0.4 | 0.004 | down |
|  | Pa_4_3858 | Unknown function | 0.07 | 0.04 – 0.10 | 0.03 – 0.12 | 0.008 | down |
|  | Pa_1_24410 | SAM  methyl transferase | 0.8 | 0.55 – 0.9 | 0.5 – 1.0 | 0.017 | N/S |
|  | Pa_5_9770 | *PAG* | 0.7 | 0.5 – 0.9 | 0.45 – 1.2 | 0.06 | N/S |
|  | Pa_3_1710 | *AOX* | 1.7 | 1.3 – 2.1 | 1.0 – 2.5 | 0 | N/S |
|  | Pa_4_3160 | *PEPCK* | 1.8 | 1.2 - 3 | 0.8 – 3.8 | 0.03 | N/S |
|  | Pa_4_80 | Methyl-transferase | 1.6 | 0.9- 3.0 | 0.8 – 3.4 | 0.12 | N/S |
| mat- | Pa_1_13940 | *PaHMG5* | 2.0 | 1.7 – 2.5 | 1.4 – 3.2 | 0.000 | up |
|  | Pa_1_14230 | *PaHMG6* | 1.3 | 1.1 – 1.4 | 1.0 – 1.5 | 0.001 | N/S |
|  | Pa_6_4110 | *PaHMG8* | 1.3 | 1.2 – 1.5 | 1.0 – 1.7 | 0.005 | N/S |
|  | Pa_7_7190 | *PaHMG9/KEF1* | 0.55 | 0.4 – 0.8 | 0.35 – 0.9 | 0.004 | down |
|  | N/A | *FMR1* | 1.2 | 1.0 – 1.5 | 0.8 – 1.7 | 0.04 | N/S |
|  | Pa_1_8290 | *MFM* | 1.2 | 1.0 – 1.4 | 0.9 – 1.6 | 0.04 | N/S |
|  | Pa_7_9070 | *PRE1* | 0.56 | 0.45 – 0.7 | 0.4 – 0.8 | 0.001 | down |
|  | Pa_6_7350 | protease | 2.2 | 1.8 – 2.7 | 1.4 - 3 | 0.003 | up |

^a^: the fold-change is the ratio of cDNA in *Δmthmg1* strain to *WT* (Materials and Methods).

^b^: transcription in *Δmthmg1* strains*.* N/S: not significant.
