# Supplementary material for: A Network of HMG-box Transcription Factors Regulates Sexual Cycle in the Fungus Podospora anserina
Source: PLoS Genet. 2013 Jul 18;9(7):e1003642. doi: 10.1371/journal.pgen.1003642 (PMC3730723; doi:10.1371/journal.pgen.1003642)
Supplement: Table S8 — Phenotype of F. graminearum strains deleted for HMGB genes (adapted from [43]). (DOC) [file pgen.1003642.s015.doc]

**Table S8.** Phenotype of *F. graminearum* strains with deleted HMG-box genes (adapted from Son et al, 2011, PLoS Pathog 7: e1002310).

| *P. anserina* gene name (gene number) | Locus ID in *F. graminearum* | *F. graminearum* gene name | Number of perithecium^a^ | Perithecium maturation^b^ | Ascospore formation^c^ | Ascospore discharge^d^ |
| --- | --- | --- | --- | --- | --- | --- |
| *PaHMG5* (*Pa_1_13940*) | FGSG_01366 | *GzHMG010* | 1 | 1 | 0 | 0 |
| *FPR1*  (*Pa_1_20590*) | FGSG_08893 | *MAT1-2-1* | 1 | 1 | 0 | 0 |
| *FMR1* | FGSG_08892 | *MAT1-1-1* | 1 | 1 | 0 | 0 |
| *SMR2* | FGSG_08890 | *MAT1-1-3* | 4 | 3 | 0 | 0 |
| *mtHMG1*  (*Pa_1_13340*) | FGSG_01201 | *GzHMG008* | 4 | 4 | 4 | 4 |
| *PaHMG2* (*Pa_1_7390*) | FGSG_07116 | *GzHMG021* | 4 | 4 | 4 | 4 |
| *PaHMG3* (*Pa_1_9380*) | FGSG_13004 | *GzHMG035* | 4 | 4 | 4 | 4 |
| *PaHMG4* (*Pa_1_11050*) | FGSG_00729 | *GzHMG005* | 2 | 2 | 4 | 4 |
| *PaHMG6* (*Pa_1_14230*) | FGSG_00385 | *GzHMG002* | 0 | 0 | 0 | 0 |
| *PaHMG7* (*Pa_5_8400*) | FGSG_07947 | *GzHMG025* | 4 | 4 | 4 | 4 |
| *PaHMG8*  (*Pa_6_4110*) | FGSG_05151 | *GzHMG016* | 4 | 4 | 4 | 4 |
| *PaHMG9*/*KEF1* (*Pa_7_7190*) | FGSG_06760 | not determined | not determined | not determined | not determined | not determined |

^a^The number of perithecia was scored from “0” to “4” (0, no perithecium; 1, less than 25% of the wild-type strain; 2, 25-50% less; 3, 50-75% less; 4, 75-100% less).

^b^Perithecium maturation was determined by size compared with the wild-type strain (4, normal; 3, 1-2 day delayed; 2, 3-7 days delayed; 1, more than 7 days delayed maturation).

^c^Ascospore formation represents the existence and morphology of ascospores (4, normal ascospores; 0, no observable ascospores).

^d^Ascospore discharge was determined according to the number of discharged ascospores compared with that of the wild-type strain (4, as wild type ; 0, no discharge).
