# Supplementary material for: A Network of HMG-box Transcription Factors Regulates Sexual Cycle in the Fungus Podospora anserina
Source: PLoS Genet. 2013 Jul 18;9(7):e1003642. doi: 10.1371/journal.pgen.1003642 (PMC3730723; doi:10.1371/journal.pgen.1003642)
Supplement: Table S9 — Oligonucleotide primers used for HMG-box gene deletion. (DOC) [file pgen.1003642.s016.doc]

**Table S9**. Oligonucleotide primers used for HMG-box gene deletion.

| Gene name (gene number) | Primer name | Primer sequence 5’>3’^a^ |
| --- | --- | --- |
| *PaHMG2* (Pa_1_7390) | 5f_7390 | cagcagccgcacgcggcgcatctcggaacggtcgacagtagttgcgtc |
|  | 5r_7390 | gtaacgccagggttttcccagtcacgacggcgcgcctgtgccgattgtaactgtac |
|  | 3r_7390 | tccttctttctagctagaggatcctctacgtcgagcccaatagaaaag |
|  | 3f_7390 | gcggataacaatttcacacaggaaacagcggcgcgcctcactcatcatctgctatc |
| *PaHMG3* (Pa_1_9380) | 5f_9380 | cagcagccgcacgcggcgcatctcggaacggtcaggggtattcaaag |
|  | 5r_9380 | gtaacgccagggttttcccagtcacgacggcgcgccgctgctgctagatgagatg |
|  | 3r_9380 | tccttctttctagctagaggatcctctacgcggtgactatcctcatctg |
|  | 3f_9380 | gcggataacaatttcacacaggaaacagcggcgcgccgctactgctatggctttgaac |
| *PaHMG4* (Pa_1_11050) | 5f_11050 | cagcagccgcacgcggcgcatctcggaacggaggtgctggaaaatgaag |
|  | 5r_11050 | gtaacgccagggttttcccagtcacgacggcgcgccggtgtctcaaagacaacag |
|  | 3r_11050 | tccttctttctagctagaggatcctctactgctcgtaagaagaccaag |
|  | 3f_11050 | gcggataacaatttcacacaggaaacagcggcgcgccggctggtcagttagcaaaac |
| *PaHMG5* (Pa_1_13940) | 5f_13940 | cagcagccgcacgcggcgcatctcggaacgcgatccatgctagaggttg |
|  | 5r_13940 | gtaacgccagggttttcccagtcacgacggcgcgcccagcccttcaagaatttc |
|  | 3r_13940 | tccttctttctagctagaggatcctctactcagtcgggcgtaaaaatg |
|  | 3f_13940 | gcggataacaatttcacacaggaaacagcggcgcgccgcttccgaatgtccacaaag |
| *PaHMG6* (Pa_1_14230) | 5f_14230 | cagcagccgcacgcggcgcatctcggaacgagagcaccatgttagtctg |
|  | 5r_14230 | gtaacgccagggttttcccagtcacgacggcgcgccgtggtgtagtaggatgtg |
|  | 3r_14230 | tccttctttctagctagaggatcctctacggagtcgtcctaaatacactc |
|  | 3f_14230 | gcggataacaatttcacacaggaaacagcggcgcgccgaggcaaggtgcatagattg |
| *PaHMG7* (Pa_5_8400) | 5f_8400 | gtaacgccagggttttcccagtcacgacggcgcgccctccgcaaaaatccacttg |
|  | 5r_8400 | cagcagccgcacgcggcgcatctcggaaccctcagcaaactactactc |
|  | 3r_8400 | gcggataacaatttcacacaggaaacagcggcgcgccggcttcacctccatcatag |
|  | 3f_8400 | tccttctttctagctagaggatcctctacgtggtggatttgacgtgatg |
| *PaHMG8* (Pa_6_4110) | 5f_4110 | cagcagccgcacgcggcgcatctcggaacgcctacaaccagaaagactag |
|  | 5r_4110 | gtaacgccagggttttcccagtcacgacggcgcgccgaacggaatgtctgcaaac |
|  | 3r_4110 | tccttctttctagctagaggatcctctacggtcggttcagtttaggatag |
|  | 3f_4110 | gcggataacaatttcacacaggaaacagcggcgcgcctggaaagaagactgatac |
| *PaHMG9*/*KEF1* (Pa_7_7190) | 5f_7190 | cagcagccgcacgcggcgcatctcggaacagtgaggtcgtccattttttg |
|  | 5r_7190 | gtaacgccagggttttcccagtcacgacggcgcgccgaggtgaggatcatcttctg |
|  | 3r_7190 | tccttctttctagctagaggatcctctacggaggatgattagagtgtc |
|  | 3f_7190 | gcggataacaatttcacacaggaaacagcggcgcgccggtgctgaaggatcagttg |

^a^: color code: red: hph; blue: pRS426; green: *Asc*I; black: target gene sequence.
