# Supplementary material for: A Network of HMG-box Transcription Factors Regulates Sexual Cycle in the Fungus Podospora anserina
Source: PLoS Genet. 2013 Jul 18;9(7):e1003642. doi: 10.1371/journal.pgen.1003642 (PMC3730723; doi:10.1371/journal.pgen.1003642)
Supplement: Table S10 — Oligonucleotide primers used for HMG-box gene amplification. (DOC) [file pgen.1003642.s017.doc]

**Table S10.** Oligonucleotide primers used for HMG-box gene amplification.

| Gene name (gene number) | Primer name | Primer sequence 5’>3’ |
| --- | --- | --- |
| *PaHMG2* (Pa_1_7390) | 5_7390 | gctccaagacagcgtaacaa |
|  | 3_7390 | cccgtcaagcggaataaaa |
| *PaHMG4* (Pa_1_11050) | 5_11050 | gcgacagaagctggttcga |
|  | 3_11050 | gagactggatgagggtggtg |
| *PaHMG5* (Pa_1_13940) | 5_13940 | aggcttccgaatgcgtcaa |
|  | 3_13940 | ccaagacacggtcccaagta |
|  | 5PSTE11 | P-gatcgcaatacccccctctc |
|  | 3HindSTE11 | tccaagcttctttcttggttctgccc |
|  | Nde13 | cacaacctctcatatggatcgcaataccccc |
|  | HisBam13 | gcggatcctcagtggtgatgatggtggtgttgatcagccttggtttcggccc |
| *PaHMG6* (Pa_1_14230) | 5_14230 | gcggtcacgatctttgttcc |
|  | 3_14230 | gggccttctggtgtagtgatat |
| *PaHMG8* (Pa_6_4110) | 5_4110 | ttgaagcgaacaagaggatg |
|  | 3_4110 | tgggctgaagaaatgggtg |
| *PaHMG9*/*KEF1* (Pa_7_7190) | 5_7190 | tgttgcgatgccattcagg |
|  | 3_7190 | cattgtggatttgtatgtgggta |
