# Supplementary material for: A Network of HMG-box Transcription Factors Regulates Sexual Cycle in the Fungus Podospora anserina
Source: PLoS Genet. 2013 Jul 18;9(7):e1003642. doi: 10.1371/journal.pgen.1003642 (PMC3730723; doi:10.1371/journal.pgen.1003642)
Supplement: Table S11 — Complementation of P. anserina mutant strains. (DOC) [file pgen.1003642.s018.doc]

**Table S11.** Complementation of *P. anserina* mutant strains.

| strain | phenotype of the complemented mutants  (number of complemented strains/ total number of co-transformants) |
| --- | --- |
|
| *ΔPahmg5* | male fertile (9/20) |
| *ΔPahmg8* | female fertile (12/30) |
| *Δkef1/ΔPahmg9* | growth and mycelium as wild type, female fertile (13/24 ^a^) |
| *ΔPahmg6* | growth and mycelium as wild type, female fertile (30/30) |
| *ΔPahmg2* | growth and mycelium as wild-type, female fertile (5/35) |
| *ΔPahmg4* | Ring as wild type, number of spermatia intermediate between wild type and mutant (2/10) |
| *ΔPahmg3* | not determined |
| *ΔPahmg7* | not determined |
| *ΔPahmg3 ΔPahmg7* | not determined |

^a^ Three transformants among the 13 with wild-type mycelium were tested for spermatia production and behaved as wild type.
