# Supplementary material for: A Network of HMG-box Transcription Factors Regulates Sexual Cycle in the Fungus Podospora anserina
Source: PLoS Genet. 2013 Jul 18;9(7):e1003642. doi: 10.1371/journal.pgen.1003642 (PMC3730723; doi:10.1371/journal.pgen.1003642)
Supplement: Table S12 — Oligonucleotide primers used for RT-qPCR. (DOC) [file pgen.1003642.s019.doc]

**Table S12.** Oligonucleotide primers used for RT-qPCR.

| Type of gene^a^ | Gene number | Gene name or function | Primer name | Primer sequence 5’>3’ ^b^ | Amplicon size |
| --- | --- | --- | --- | --- | --- |
| HMGB | *Pa_1_13340* | *mtHMG1* | 13340f | GAGTGAGACTGAACGCCAG/CC | 130 bp |
|  |  |  | 13340r3 | TGTGATGGAGGGCTCAAAA |  |
| HMGB | *Pa_1_13940* | *PaHMG5* | 13940AF2 | CGGCCCTATTCTAGAT/ATGTC | 187 bp |
|  |  |  | 13940intR | CGGCTTCGCTCTTTCCTTC |  |
| HMGB | *Pa_1_14230* | *PaHMG6* | 14230f | CTTAGGAGCGTTGGGGT/CCTTC | 98 bp |
|  |  |  | 14230r | GATTCACATCAATCATTATGCCTA |  |
| HMGB | *Pa_6_4110* | *PaHMG8* | 4110f | ACGCTGTCGGATAAGTTTGC | 190 bp |
|  |  |  | 4110r | CCTGTTGCTGCTGCTCCTT |  |
| HMGB | *Pa_7_7190* | *KEF1/PaHMG9* | 7190f | ACCGACTGTAAGTTCATCACCAT | 200 bp |
|  |  |  | 7190r | CACTCCCATCAACAGGCAAA |  |
| HMGB | *Pa_1_20590* | *FPR1* | FPR1f | GGCGTTCTCAATACAATGAA/GTCG | 297 bp |
|  |  |  | FPR1r | GCCACCAGTCATGACGGAAATG |  |
| GoI | *Pa_2_2310* | *MFP* | MFPf | CGTACGGGAGTGGACTTGGATGGA | 149 bp |
|  |  |  | MFPr | CGACACTGAGGCGGTACCCAAAAG |  |
| GoI | *Pa_4_1380* | *PRE2* | 1380f | GTTGATGTTTGTGCCCG/TGG | 177 bp |
|  |  |  | 1380r | TCGGTGGTTGTGCCAGTCG |  |
| GoI | *Pa_4_3858* | *3858* | 3858f | CTTTCCTGACGATTC/GACC | 171 bp |
|  |  |  | 3858r | CAAGTGTATTGCTTGTGGGTT |  |
| GoI | *Pa_1_24410* | SAM | 24410f | GCACCATCACCAAAGCAATC | 151 bp |
|  |  |  | 24410r | TCTTCAAAGGGGAGTTT/CACA |  |
| GoI | *Pa_5_9770* | PAG | 9770f | ATGAAGCGCAGCAGGATGT | 147 bp |
|  |  |  | 9770r | TGTCAAAGATGACTTTG/AATCTC |  |
| GoI | *Pa_3_1710* | *AOX* | 1710f | CTGGTCCGTCTGTGTCG/ATGG | 139 bp |
|  |  |  | 1710r | AAATGAACCGAACAAG/CCATT |  |
| GoI | *Pa_4_3160* | *PEPCK* | 3160f | CCGAGAACGAAATCTG/GTGG | 196 bp |
|  |  |  | 3160r | ATGAAGAGGGCATGGTAGGC |  |
| GoI | *Pa_4_80* | Methyl transferase1 | 80f_I1 | GCTTACATTATGCTCTT/TGGC | 209 bp |
|  |  |  | 80r_I1 | CCGTTGGTTCCGTTCTCAC |  |
| HMGB | N/A^c^ | *FMR1* | FMR1f | GGTTTCATGGGCTACCGAT/CCTAC | 251 bp |
|  |  |  | FMR1r | CATCCAAGGGCTTCCATGTAGC |  |
| GoI | *Pa_1_8290* | *MFM* | MFMf | CCACCCTCGCAACAACACGTTAGA | 150 bp |
|  |  |  | MFMr | AAACGAAGGCGATGCTCATGTTGG |  |
| GoI | *Pa_7_9070* | *PRE1* | 9070f | CGGCGGTCATCTTTACGGT | 195 bp |
|  |  |  | 9070r | GGTAAAAGGTGAGGC/AAGCC |  |
| GoI | *Pa_6_7350* | protease | 7350f | AAGTTCCCTGCTCGATGGT | 205 bp |
|  |  |  | 7350r | GTTTGGTCAAACACGA/CATAC |  |
| HKG | *Pa_1_16650* | *AS1* | AS1f | CAACATGGCTGACGAATAC/AACGC | 115 bp |
|  |  |  | AS1r | GGAGGTCAGGTCAAGGAGA/GCATC |  |
| HKG | *Pa_3_6780* | *CIT1* | CIT1f | CTCCTCCAAGACCCAG/ACCCTC | 100 bp |
|  |  |  | CIT1r | GACCTTGGAGCCATGCTCC/TTTC |  |
| HKG | *Pa_3_5110* | *GPD* | GPDf | CATTGAGCCCAAGTACGCT/GAG | 113 bp |
|  |  |  | GPDr | GTCGCGCTCAGTGTAGAACTTGA |  |
| HKG | *Pa_5_5390* | *H2A* | H2Af | GCAAGAACGCGCAATC/TCGTTC | 329 bp |
|  |  |  | H2Ar | AGTCTTCTTGGGAAGAAGGT/TCTG |  |
| HKG | *Pa_7_8770* | *LEU1* | LEUf | CACTGCGGTCCGGAG/GTTGTTG | 126 bp |
|  |  |  | LEUr | GTGGGCGGTGATGGAGGC/ACC |  |
| HKG | *Pa_2_6460* | *PAH1* | PAH1f | GATCTGGTTCCAGAACCG/ACGTG | 247 bp |
|  |  |  | PAH1r | GCAGTTGAGATGATGAATCA/AAAC |  |
| HKG | *Pa_7_6690* | *PDF2* | PDFf | GCAGACAGGTTCGAAAAG/ATTG | 294 bp |
|  |  |  | PDFr | CAGATGATCAATGGTT/TCTTGC |  |
| HKG | *Pa_4_8980* | *TBP* | TBPf | CACACCCACTCTTCA/GAACATT | 106 bp |
|  |  |  | TBPr | ACGCTTGGGGTTGTA/CTCAGC |  |
| HKG | *Pa_7_8490* | *TIP41* | TIPf | GTTTGCGGAGGTGAAGAAG/AA | 146 bp |
|  |  |  | TIPr | CCGTCTCACCCTCGAGAC |  |
| HKG | *Pa_4_7790* | *UBC* | UBCf | GGCCATCCCCATCCATCAAC | 107 bp |
|  |  |  | UBCr | GGTGATGGTCTTGCCAGTGA/GA |  |

^a^: HKG: housekeeping gene, used as candidate for reference gene; GoI: gene of interest, HMGB: HMG-box gene.

^b^: slashes (/) separate sequences on two consecutive exons.

^C^: N/A not applicable.
