# Supplementary material for: A Network of HMG-box Transcription Factors Regulates Sexual Cycle in the Fungus Podospora anserina
Source: PLoS Genet. 2013 Jul 18;9(7):e1003642. doi: 10.1371/journal.pgen.1003642 (PMC3730723; doi:10.1371/journal.pgen.1003642)
Supplement: Table S13 — Normalization genes (REF) used for RT-qPCR. (DOC) [file pgen.1003642.s020.doc]

**Table S13.** Normalization genes (REF) used for RT-qPCR.

| Gene name (gene number) | Strain *ΔPahmg5* | | Strain *ΔPahmg6* | | Strain *ΔPahmg8* | | Strain *Δkef1* (*ΔPahmg9)* | | Strain *Δmthmg1* | |
| --- | --- | --- | --- | --- | --- | --- | --- | --- | --- | --- |
|  | *mat+* | *mat-* | *mat+* | *mat-* | *mat+* | *mat-* | *mat+* | *mat-* | *mat+* | *mat-* |
| *AS1* (Pa_1_16650) | REF | REF | REF | REF | REF | REF | REF | REF | REF | - |
| *CIT1* (Pa_3_6780) | - | - | - | - | - | - | - | - | - | REF |
| *GPD* (Pa_3_5110) | - | - | - | - | REF | REF | - | - | - | REF |
| *H2A* (Pa_5_5390) | REF | REF | - | - | - | - | - | - | - | - |
| *LEU1* (Pa_7_8770) | - | - | - | - | - | - | - | - | - | - |
| *PAH1* (Pa_2_6460) | - | - | - | - | - | - | - | - | - | - |
| *PDF2* (Pa_7_6690) | - | - | - | - | - | - | - | - | REF | REF |
| *TBP* (Pa_4_8980) | REF | REF | - | - | - | - | - | - | - | - |
| *TIP41* (Pa_7_8490) | REF | REF | - | - | REF | REF | - | - | - | - |
| *UBC* (Pa_4_7790) | - | - | - | - | - | - | - | - | REF | - |
