# Supplementary material for: A Network of HMG-box Transcription Factors Regulates Sexual Cycle in the Fungus Podospora anserina
Source: PLoS Genet. 2013 Jul 18;9(7):e1003642. doi: 10.1371/journal.pgen.1003642 (PMC3730723; doi:10.1371/journal.pgen.1003642)
Supplement: Table S14 — Oligonucleotides primers used for EMSA. (DOC) [file pgen.1003642.s021.doc]

**Table S14.** Oligonucleotide primers used for EMSA.

| Gene name or function | Gene number | Primer name | Primer sequence 5’>3’ ^a^ |
| --- | --- | --- | --- |
| *KEF1/PaHMG9* | Pa_1_7190 | W7190_2 | GATCGTAGCTATAGACAAAGAAAGCAACAGATAG |
|  |  | C7190-2 | GCTATCTGTTGCTTTCTTTGTCTATAGCTACGAT |
| *mtHMG1* | Pa_1_13340 | W13340_2 | GGAGGGCATCTTCAACAAAGAATCTCGGCAAATA |
|  |  | C13340_2 | GTATTTGCCGAGATTCTTTGTTGAAGATGCCCTC |
| *PaHMG5* | Pa_1_13940 | W13940_2 | GTAGAGAAGCCGGAACAAAGAAAGGAACAAGCAG |
|  |  | C13940_2 | GCTGCTTGTTCCTTTCTTTGTTCCGGCTTCTCTA |
| *PaHMG8* | Pa_6_4110 | W4110_2 | GTGCAAACGCGGGGACAAAGAAAAAGTCTGGTGC |
|  |  | C4110_2 | GGCACCAGACTTTTTCTTTGTCCCCGCGTTTGCA |
| *FMR1* | N/A | WFMR1_2 | GTGCAGGATAGCCAACAAAGAAATGACTGCCACG |
|  |  | CFMR1_2 | GCGTGGCAGTCATTTCTTTGTTGGCTATCCTGCA |
| *FPR1* | Pa_1_20590 | WFPR1_3 | GGTGAAGGTGAATAACAAAGAAATACACTGACGG |
|  |  | CFPR1_3 | GCCGTCAGTGTATTTCTTTGTTATTCACCTTCAC |
| *PaHMG2* | Pa_1_7390 | W7390_1 | GTCGGTAGGCGTGGACAAAAACAACACAGTGGAC |
|  |  | C7390_1 | GGTCCACTGTGTTGTTTTTGTCCACGCCTACCGA |
| *PaHMG3* | *Pa_1_9380* | W9380_1 | GGATTGGAATAGAAACCAAGACAAGACACCTCAA |
|  |  | C9380_1 | GTTGAGGTGTCTTGTCTTGGTTTCTATTCCAATC |
| *PaHMG4* | Pa_1_11050 | W11050_1 | GTCAAAATCAATCAGCAAAGAAAAAAATCACCCC |
|  |  | C11050_1 | GGGGGTGATTTTTTTCTTTGCTGATTGATTTTGA |
| *PaHMG6* | Pa_1_14230 | W14230_1 | GCTCATGCCTGGTAACAAAAAAAAGCTGCAGCCG |
|  |  | C14230_1 | GCGGCTGCAGCTTTTTTTTGTTACCAGGCATGAG |
| *MFM* | Pa_1_8290 | WMFM_2 | GACAAGACAGATCATCAAAGACACGATGTGACCG |
|  |  | CMFM_2 | GCGGTCACATCGTGTCTTTGATGATCTGTCTTGT |
| *PRE1* | Pa_7_9070 | WPRE1_2 | GCGGCCTGGCATCAACAAAGACCGGCGGGCCAGC |
|  |  | CPRE1_2 | GGCTGGCCCGCCGGTCTTTGTTGATGCCAGGCCG |
| protease | Pa_6_7350 | W7350_1 | GACGAAATCTAAAAACAAAGACATAAAGAGATTG |
|  |  | C7350_1 | GCAATCTCTTTATGTCTTTGTTTTTAGATTTCGT |
| MFP | Pa_2_2310 | WMFP_2 | GTCATCTCCAAGGATCAAAGAAACCAATTTGGTC |
|  |  | CMFP_2 | GGACCAAATTGGTTTCTTTGATCCTTGGAGATGA |
| *SAM methyl transferase* | Pa_1_24410 | W24410_1 | GATCGTAAGTCTTAACAAAGAACGCTGATCCATG |
|  |  | C24410_1 | GCATGGATCAGCGTTCTTTGTTAAGACTTACGAT |
| *AOX* | Pa_3_1710 | WAOX_1 | GCCCAGAAACCTCGACAAAGAATACCAACCCCGA |
|  |  | CAOX_1 | GTCGGGGTTGGTATTCTTTGTCGAGGTTTCTGGG |
| PEPCK | Pa_4_3160 | WPEPCK_1 | GTTTGTTTGGTGCGACAAAGATACATCCCAATCC |
|  |  | CPEPCK_1 | GGGATTGGGATGTATCTTTGTCGCACCAAACAAA |
| FMR1 scrambled | N/A | WFMR1Sc_2 | GGCGTAACGGTAGCTAGGATAACCACAACGACAA |
|  |  | CFMR1Sc_2 | GTTGTCGTTGTGGTTATCCTAGCTACCGTTACGC |

^a^: color code: red: putative HMG-box binding site (see Figure 7).
